# Environmental Quality Assessment Using Fecal Metabolomics in Waterfowl from Protected Wetlands in Southwest Spain

**DOI:** 10.1021/acsestwater.5c01312

**Published:** 2026-02-10

**Authors:** Cristina Navarro-Fernández, Belén Callejón-Leblic, Ángel Rafael Domínguez-Bustos, Isabel Molina, Francisco Hortas, Tamara García-Barrera

**Affiliations:** † Research Center for Natural Resources, Health and the Environment (RENSMA). Department of Chemistry, Faculty of Experimental Sciences, 16743 University of Huelva, Campus El Carmen, Fuerzas Armadas Ave., Huelva 21007, Spain; ‡ Andalusian Network of Centres for the Recovery of Threatened Species, CREA Dunas de San Anton, Camino de los Enamorados s/n, El Puerto de Santa María 11500, Spain; § Department of Biology, Institute of Marine Research (INMAR), University of Cadiz, Puerto Real 11510, Spain

**Keywords:** waterfowl, Odiel Marshes Biosphere Reserve, Cadiz Bay IBA, fecal metabolomics

## Abstract

The study of environmental stress requires an understanding of biological responses to pollutants, which can be difficult to interpret due to multiple influencing factors. This study investigates the fecal metabolome of waterfowl as bioindicators of environmental quality in two protected wetlands in southwestern Spain: the Odiel Marshes Biosphere Reserve (OMBR) and the Cádiz Bay Important Bird Area (CBIBA). Using untargeted UHPLC-QTOF-MS metabolomics, fecal samples were analyzed from five species: spoonbills, black-headed gulls, yellow-legged gulls, lesser black-backed gulls, and storks. Significant alterations were observed in key metabolites, including fatty acids, steroids, glycerophospholipids, and sphingolipids, reflecting differences in diet, habitat use, and pollutant exposure. In spoonbills, steroids and fatty acids represented the largest fractions of the detected metabolites, while comparisons between gull species revealed variations in fatty acid and glycerophospholipid levels. Multivariate analyses showed a clear separation between species and sites. Pathway analysis identified several altered metabolic routes, primarily involving fatty acid, bile acid, and sphingolipid metabolism. Overall, these results demonstrate that fecal metabolomics is a robust noninvasive tool for assessing pollution-related physiological changes, confirming waterbirds as effective bioindicators of wetland ecosystem health.

## Graphical abstract

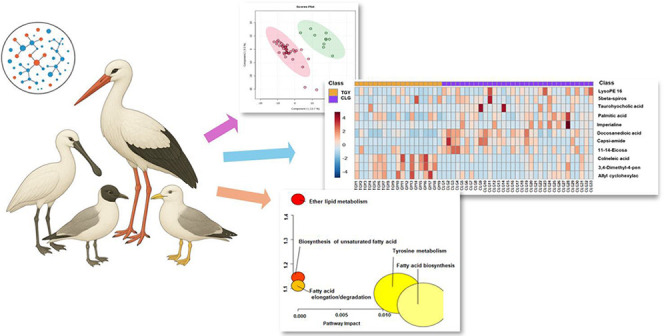

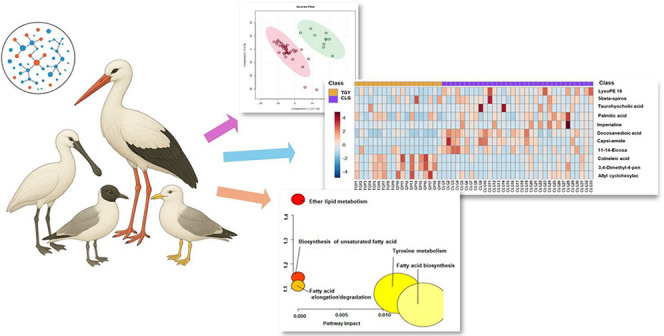

## Introduction

The Odiel Marshes Biosphere Reserve (OMBR) in the Huelva Estuary and Cadiz Bay IBA (CBIBA) are two protected wetlands of southwestern Spain. The CBIBA is mostly occupied by the Cadiz Bay Natural Park and other saltworks like Cetina. The OMBR ecosystem has been historically more contaminated,^1^ affected by acid mine drainage (AMD), chemical industries, and releases of pollutants from five phosphoric acid industrial plants and phosphogypsum waste stacks,^2^ resulting in some areas where vegetation is unable to thrive.^3^ The CBIBA is also affected by AMD and the discharge of the Guadiana and the Guadalquivir rivers.^4,5^ Nevertheless, in the OMBR, a greater number of pollutants tend to accumulate because it is a more closed system that is also affected by the river discharge.^6^ In contrast, the Cetina and La Tapa saltworks in CBIBA have less pollution due to the more direct connection with the Atlantic Ocean, allowing the renewal and dispersion of pollutants. Although the environmental levels of different pollutants in water and sediments from the OMBR^7–9^ and CBIBA^10–12^ have been extensively reported, the potential biological response of waterfowl has been rarely studied. The study of environmental stress situations requires a deep understanding of the biological response to pollutants, which can be challenging to interpret due to the myriad factors influencing them and the complex processes of synergism and antagonism between pollutants.^13^ Waterbirds bioaccumulate pollutants from water, sediments, and their prey.^14–16^ Their biological responses, including physiological alterations and oxidative stress biomarkers, directly reflect environmental contamination.^17^ Dependent on aquatic habitats, waterbirds are highly sensitive to changes in water quality and habitat conditions.^14^ Chronic pollutant exposure can compromise their health and reproductive performance.^16^ Monitoring waterbirds provides early warning of environmental problems, enables ecological risk assessment, and informs conservation management decisions.^17,18^ Moreover, these species serve as effective indicators of habitat condition and overall aquatic ecosystem quality.^18^ In this sense, metabolomics holds a significant promise providing crucial insights of metabolites in free-living animals that participate in metabolic reactions for growth, maintenance, and response to stressors.^19^ Among biological samples used, feces have been used as a proxy for measuring dietary contaminant exposure levels^20^ and are of great interest due to their relationship with the gut microbiota that can produce some of these metabolites.^21^ The analysis of fecal metabolites offers valuable insights into individual behavior and physiology and can be used to assess environmental pollution.^22^ Likewise, there are fecal metabolomic studies in birds that assess the impact of various factors such as season,^23^ carrion-feeding habits,^24^ climate,^25^ and food shortages.^19^ Moreover, the relationships between heavy metals in the blood and metabolic changes in the migratory pink-footed goose have been reported.^26^ However, there is a lack of research using fecal metabolomics in birds as bioindicators to assess environmental pollution and its potential biological effects, along with other factors such as feeding or migration patterns.

Here, we evaluated the potential biological impact caused by the differential pollution of OMBR and CBIBA wetlands by means of fecal metabolomics in waterfowl used as bioindicators.

## Material and Methods

### Chemicals and Reagents

Methanol, acetonitrile (LC-MS grade), and formic acid were supplied by Fisher Scientific (Leicestershire, UK). Water was purified with a Milli-Q Gradient system (Millipore, Watford, UK). Internal standard d_31_-palmitic acid was purchased from Sigma-Aldrich (Steinheim, Germany). Canvax stool sample collection stabilization kit SC0012S was supplied by Canvax Reagents SL (Spain).

### Sampling Sites

In this study, two protected wetlands situated in southwestern Spain, specifically in Andalusia, were examined. Fecal samples were collected from the OMBR as well as from two sites in the CBIBA called the Cetina and La Tapa saltworks. OMBR, covering 7000 ha, comprises different biotypes, such as dune bars, high marshes, and middle marshes. This ecosystem is inside the Huelva Estuary, formed by the common mouths of the Odiel and Tinto Rivers. The traditional saltworks in the CBIBA were already established in ancient times because of the geographical characteristics and the ideal climate.^27,28^ The GPS coordinates of each of the locations are detailed in Figure S1 of the Supporting Information.

### Waterfowl Species Used in the Study

Five different waterfowls were selected as bioindicators of the study areas: V Eurasian spoonbill (*Platalea leucorodia*), the lesser black-backed gull (*Larus fucus*), the yellow-legged gull (*Larus michahellis*), the black-headed gull (*Chroicocephalus ridibundus*), and the white stork (*Ciconia ciconia*). In our study, fecal samples were divided into distinct groups based on waterfowl species and the sampling location. Samples of spoonbills were designated as *OS* and *CS* in the OMBR and CBIBA, respectively. Similarly, the *OG* group comprised samples from black-headed gulls in the OMBR, whereas the *TYG* and *TS* groups correspond to fecal samples from yellow-legged gulls and storks, respectively, taken at La Tapa Saltworks of the CBIBA. Finally, samples of lesser black-backed gulls from the Cetina Saltworks of the CBIBA were designated as the *CLG* group.

The study was approved by the ethical committee of the Universities of Huelva (CEEA-UHU-0042024) and Cádiz (23/05/2024), and by the Directorate-General for Agricultural and Livestock Production of the Regional Ministry of Agriculture, Fisheries, Water and Rural Development (code num. ES110120000210), as well as by the Andalusian Ministry of Sustainability and Environment (Spain) (Code num. EXT/2024/0000000000938674).

### Sample Collection

Feces (*n* = 176) were collected in the habitats where the species were resting, taking care that they were monospecific flocks of birds that were previously located with binoculars. Only fresh feces were collected. The sampling was conducted from October 2023 to November 2023. Regarding the morphology and composition of the samples, no differences were found in the feces. These features can change depending on the diet, but no unusual results were observed. Therefore, the composition and morphology were within the expected range. Since black-backed gull flocks may be mixed with yellow-legged gulls, they were considered a monospecific flock when ≥90% of individuals were black-backed gull or yellow-legged gull. Specifically, the number of fecal samples was as follows: black-headed gull (*n* = 34), yellow-legged gull (*n* = 19), white stork (*n* = 42), spoonbill (*n* = 48), and black-backed gull (*n* = 33). Table S1 describes the number of samples collected per area.

Fecal samples were collected in specific tubes with a spatula and stabilizing solution, and potential contamination with soil was avoided by taking samples in the fecal core. Feces were collected from each species at a minimum distance of two m to ensure that they belonged to different individuals. In addition, feces were collected from monospecific groups that were monitored from a distance by using a telescope. Once located, only fresh feces were collected, ensuring that samples from different waterfowl species were clearly distinguished and were not contaminated by droppings from other bird species. All samples were kept in the cold using a cold fridge with ice blocks until they reached the laboratory. They were then frozen at −80 °C until further processing.

### Sample Treatment

The fecal matter was lyophilized before extraction of the metabolites. Then, 250 μL of methanol (MeOH) was added to 10 mg of the lyophilized sample, and the mixture was vortexed for 30 min. The mixtures were centrifuged at 2057*g* and 4 °C for 10 min, and the resulting supernatant was collected and dried using a speed vacuum system (Thermo Scientific Savant SPD111 V SpeedVac Concentrator) for 30 min at 45 °C. Samples were stored at −80 °C until the analysis.

### UHPLC-QTOF-MS Analysis

Untargeted metabolomics was carried out into an ultrahigh performance liquid chromatograph coupled to a quadrupole time-of-flight mass spectrometer (UHPLC-QTOF-MS) model Agilent 1290 Series LC pump equipped with a Wellplate Autosampler and coupled to an Agilent 6550 iFunnel QTOF LC/MS System with a dual electrospray ion source that operated in negative and positive modes (Agilent Technologies, Tokyo, Japan). An inverse phase chromatography with a gradient was performed to separate the metabolites. Water (A) and acetonitrile (B) with 0.1% formic acid were used as the mobile phases. A flow rate of 0.4 mL min^–1^ running in a gradient method from 5% to 100% of phase B was selected for the analysis. Thus, 10 μL of extracted fecal samples was injected into an Agilent Zorbax Eclipse Plus C18 (20 × 3 mm; 1.8 μm; Agilent Technologies) thermostated at 60 °C. For mass correction, the reference pairs of masses (*m*/*z*) 121.0509 and 922.0098, 112.9856 and 1033.9881 were constantly introduced into the system for positive and negative ionization modes, respectively. The full scan mode was monitored from 50 to 1100 *m*/*z*. The QTOF parameters were set to 3 kV for the capillary voltage, 12 L min^–1^ at 250 °C for the drying gas flow rate, and 52 psi for the gas nebulizer. The fragmentor voltage was set to 175 V in positive and 250 V in negative ionization modes. A list containing the most significant features was imported and analyzed with the initial chromatographic conditions using Agilent MassHunter Data Acquisition software in Targeted MS/MS mode with an MS/MS scan rate of 1 spectrum s^–1^. Nitrogen was used as a collision gas, and several collision voltages were fixed from 10 to 40 V for the fragmentation of compounds. Data were acquired in centroid mode at a scan rate of 1.0 spectra per second.

### Data Processing

For UHPLC-QTOF-MS, raw data processing was conducted with Agilent MassHunter Profinder B.10.0 software (Agilent Technologies). To extract the data, the Batch Recursive Feature Extraction (RFE) wizard for small molecules in the software was applied. RFE performs two algorithms: First, the Molecular Feature Extraction algorithm (MFE) including extraction, selection of ion species, and charge state was used to find the features in the data set. Second, the initial features were aligned by the retention time (RT) and mass, creating a list of unique features through binning. Then, the RT and mass data pairs of the aligned and binning features were used as input criteria to find the features using the Find by Ion algorithm (FbI) more accurately. Additional scoring, integration, and peak filters were also applied to the data set. Moreover, Mass Profiler Professional B.10.0 (Agilent Technologies) was used for the normalization of the data set using total area sums. Table S2 shows the parameters and filters used for the positive and negative modes.

### Statistical Analysis

Mass Profiler Professional B.10.0 (Agilent Technologies) was used for the determination of the relevant metabolites. In this sense, principal component analysis (PCA) and partial least-squares discriminant analysis (PLS-DA) were conducted to compare the fecal metabolomic profiles obtained. To ensure stability and reliable metabolomics results, we evaluated the analysis using 10 quality control samples. Additionally, we calculated the coefficient of variation (CV) for the quality control samples, and only compounds with CV lower than 15% were included in the study. The software supplied the predictive and class separation parameters *R* ^2^ and *Q* ^2^ of all models built, and these are shown in Table S3. Before performing statistical analysis, the data were submitted to Pareto scaling and logarithmic transformation. One-way ANOVA and the Tukey test for multiple comparisons were applied using Mass Profiler Professional B.10.0 (Agilent Technologies). Moreover, a Benjamini–Hochberg FDR correction was also applied to adjust the p-values. The level of statistical significance for all tests was set to *p* < 0.05. We evaluated the altered metabolic pathways by using the available web tool MetaboAnalyst 6.0 (metaboanalyst.ca).

### Annotation of Fecal Metabolites

The compounds in the data set were annotated using the Agilent Qualitative Analysis Workflow MassHunter B.08.00 software. Specifically, the “Compound Discovery” workflow and “Find by Molecular Features” compound mining algorithms were employed for this purpose. To annotate the compounds, we consulted the METLIN (http://metlin.scripps.edu) and HMDB databases, focusing on compounds with a match score higher than 90%, indicating a strong correlation with the target compound in terms of mass, isotope pattern, and retention time. Additionally, MS/MS experiments were conducted using a QTOF under the same chromatographic conditions as those for the primary analysis to confirm the annotation of certain compounds. Collision-induced dissociation (CID) fragmentation was used to target ions based on their accurate mass and retention time.

## Results

### Evaluation of the Fecal Metabolome Profile of the Spoonbill Platalea leucorodia in Two Different Natural Areas

A good classification of the studied groups was observed, suggesting a differential fecal metabolome due to the habitat. Therefore, Figure 1A illustrates a heatmap of the abundance of the most important metabolites altered when comparing OS and CS (22 in total, Tables S4 and S5). Regarding the abundance of each family, several metabolites were altered, namely, carboxylic acids (4%), diazines (5%), fatty acyls (18%), glycerophospholipids (9%), macrolides, benzene derivatives (9%), prenol lipids (9%), sphingolipids (9%), and steroids (32%) (Figure 1B). The results obtained demonstrated decreased levels of fatty acyl, prenol lipids, sphingolipids, and steroids in the OS group compared to those in CS. Specifically, the levels of the fatty acids 2-hydroxy myristic acid, 10-hydroxystearic acid, 16-hydroxy hexadecanoic acid, and palmitic acid decreased significantly. Additionally, the levels of steroids and steroid derivatives such as the lithocholic acid glycine conjugate, 3,7-dihydroxy-5-cholanoic acid, 5-cyprinol sulfate, 3,6,7-trihydroxy-5-cholanoic acid, and 5-alpha-pregnan-3,20-dione as well as the ceramides Cer (d18:0/14:0) and Cer (d18:1/12:0) also decreased. A reduction in the levels of the prenol lipids phytal and 5,8-epoxy-5,8-dihydro-3-hydroxy-8′-apo-b, y-carotenal and the benzene derivatives 2-dodecylbenzenesulfonic acid and *N*-undecylbenzenesulfonic acid was observed. In contrast, when comparing OS with CS, increased levels of carboxylic acids, diazines, and macrolides such as L-isoleucyl-l-proline, inosine 3′:5′-cyclic monophosphate (cIMP), epothilone C, and the bile acids tauroursodeoxycholic acid and taurohyocholic acid were found. Concerning the alteration of phospholipids, decreased levels of phosphocholine (PC) PC (22:2/14:1) and increased levels of lysophosphoetaholamine (LPE) LPE (20:0) in OS vs CS were observed. Otherwise, the levels of other less altered metabolites of the carbohydrates and carbohydrate conjugate families, such as carboxylic acid derivatives and ceramides, decreased in OS compared to the CS group. The pathway analysis (Figure 1C and Table S6) showed six altered routes including taurine and hypotaurine metabolism, sphingolipid metabolism, biosynthesis of unsaturated fatty acids, fatty acid degradation, primary bile acid biosynthesis, and fatty acid biosynthesis.

**Figure 1:**
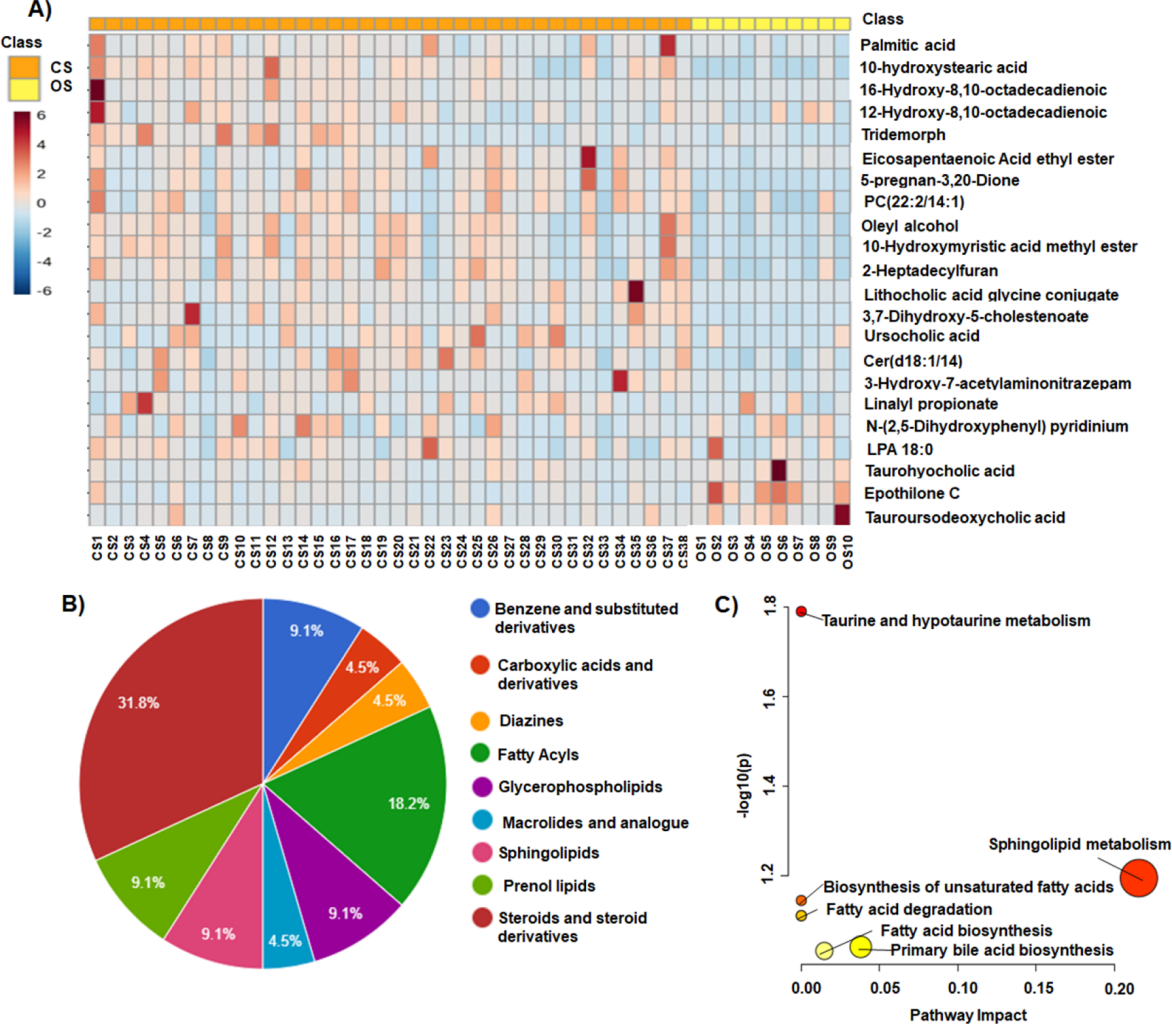
Comparison of fecal metabolomes between the OS and CS groups. (A) Heatmap representing the relative abundance of significantly altered metabolites. (B) Main classes of metabolites that were altered between the groups. (C) Metabolic pathways affected by these alterations. CS: Spoonbills from Cetina Saltworks in the CBIBI; OS: Spoonbills from the Odiel Marshes Biosphere Reserve.

### Evaluation of the Fecal Metabolome Profiles from Different Waterfowl Species within a Single Natural Habitat

A total of 37 metabolites (OS vs OG, Figure 2A) and 34 metabolites (TS vs TYG, Figure 2B) were significantly altered, and their abundances are summarized in heatmaps. Other information about the metabolites identified is collected in Tables S7 and S8.

**Figure 2:**
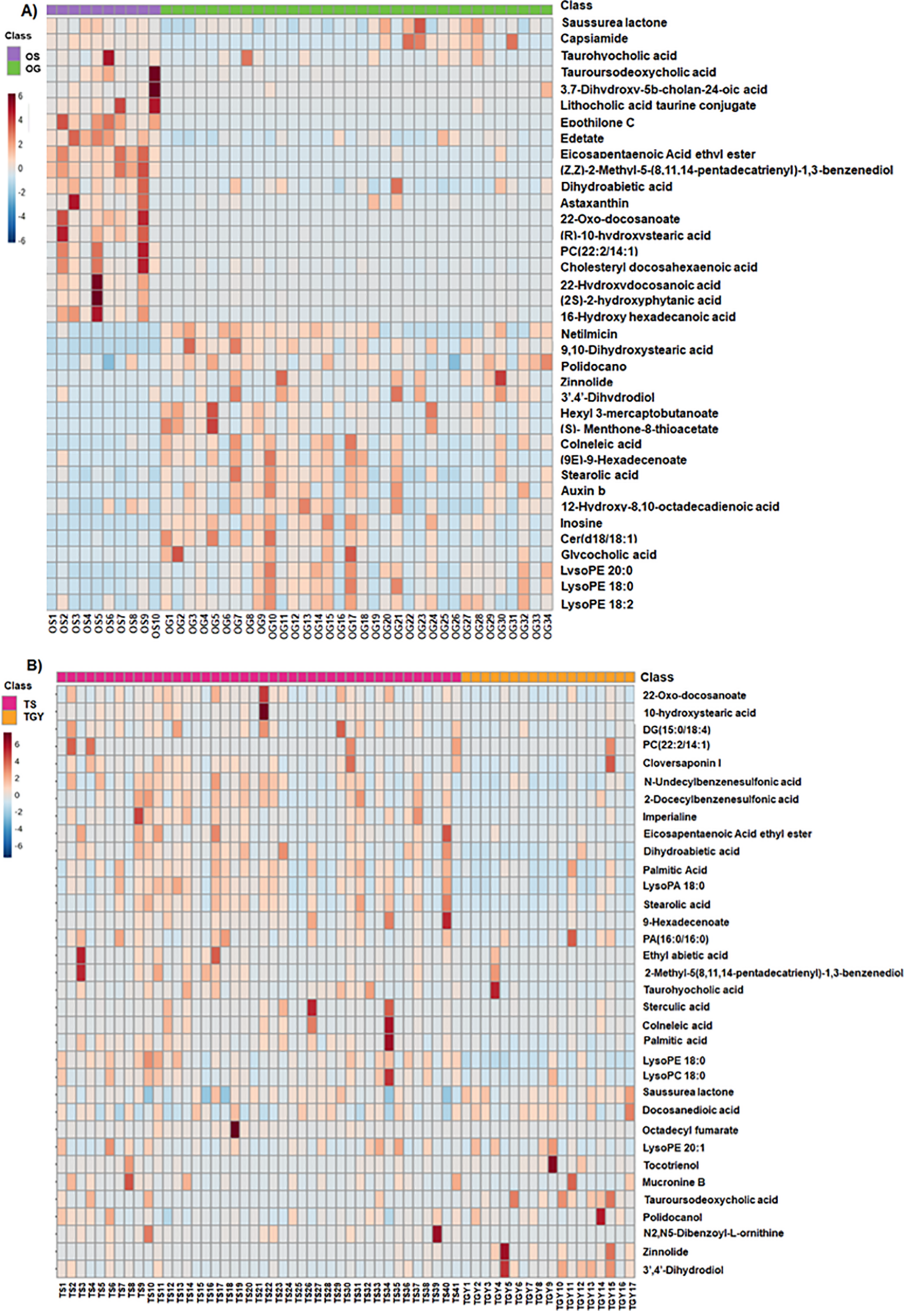
Abundance heatmap of the fecal metabolome. (A) Heatmap representing the relative abundance of significantly altered metabolites for OS and OG groups. (B) Heatmap representing the relative abundance of significantly altered metabolites for TS and TYG groups.

Significant changes in the levels of the following families of compounds were observed: amino acids, fatty acyls, glycerophospholipids, prenol lipids, and bile acids in both waterfowl fecal metabolome comparisons (OS vs OG; TYG vs TS) (Figure 2). These findings suggest that different species are affected in distinct ways by their natural habitats. Figure S2 shows the percentage of the most altered classes of annotated metabolites in the fecal metabolome of (a) OS vs OG groups (Figure S2A) and (b) TS vs TYG groups (Figure S2B). Specifically, altered fatty acyls accounted for 32% of the metabolites in OS vs OG and 36% in TS vs TYG. Prenol lipids were the second most altered class of metabolites, representing 16% in OS vs OG and 15% in TS vs TYG. However, steroids and derivatives were the third most altered group of metabolites in OS vs OG, while glycerophospholipids were the third most altered in TS vs TYG. A decrease in certain fatty acyl levels for the OS group compared to the OG was found, such as stearolic acid, 3,4-dimethyl-5-pentyl-2-furan-heptanoic acid, 9-hexadecenoate, 9,10-dihydroxy stearic acid, 12-hydroxy-8,10-octadecadienoic acid, and colneleic acid. However, the fatty acyls 10-hydroxystearic acid, 16-hydroxy hexadecanoic acid, and 22-hydroxydocosanoic exhibited significant increases in the same comparison. In the case of prenol lipids, there was also a notable increase in the levels of the monoterpenoid menthone 8-thioacetate, the diterpenoids dihydroabietic acid, 2-hydroxyphytanic acid, and saussurea lactone, as well as the tetraterpenoid astaxanthin. Moreover, there was a predominant increase in the levels of steroids and derivatives such as the bile acids 3,7-dihydroxy-5-cholan-24-oic acid, tauroursodeoxycholic acid, and taurohyocholic acid and the steroid ester cholesteryl docosahexaenoic acid, while glycocholic acid decreased. Finally, the glycerophospholipids were mainly diminished in OS compared with OG. Thus, LPE (18:2), LPE (18:0), and PC (22:2/14:1) decreased significantly, although LPE (20:0) increased.

On the other hand, when comparing TS vs TYG in the La Tapa Saltworks, there was a significant decrease of fatty acyls including octadecyl fumarate, eicosapentaenoic acid ethyl ester, 9-hexadecenoate, sterculic acid, stearolic acid, colneleic acid, 10-hydroxystearic acid, docosanedioic acid, 22-Oxo-docosanoate, palmitic acid, palmitic amide, and DG (15:0/18:4). Prenol lipids including dihydroabietic acid and ethyl abietic acid, tocotrienol, and cloversaponin also decreased in the TS group compared to the TYG. Additionally, several glycerophospholipids such as lysophosphatidic acid (LPA) LPA (18:0), the phosphatidic acid (PA) PA (16:0/16:0), and the lysophospholipids LPC (18:0), LPE (18:0), and LPE (20:1) also decreased significantly in the TS group. Levels of other less altered metabolites, including amino acids such as bencesulfonic acids and bile acids, also decreased in the TS group compared to the TYG (Table S7).

The pathway analysis showed five altered routes for OS vs OG groups (Figure S3A and Table S9) including primary bile acid biosynthesis, taurine and hypotaurine metabolism, sphingolipid metabolism, steroid biosynthesis, and purine metabolism. Additionally, the affected metabolic pathways in TS vs TYG groups (Figure S3B and Table S9) include taurine and hypotaurine metabolism, glycerolipid metabolism, ether lipid metabolism, biosynthesis of unsaturated fatty acid, fatty acid degradation, primary bile acid, and fatty acid biosynthesis.

### Evaluation of the Fecal Metabolome Profile of the Lesser Black-Backed and Yellow-Legged Gulls in La Tapa and Cetina Saltworks

A total of 11 metabolites were significantly altered in the fecal metabolome of TYG and CLG groups that are summarized in the heatmap of abundance (Figure 3A, Tables S10, and S11).

**Figure 3:**
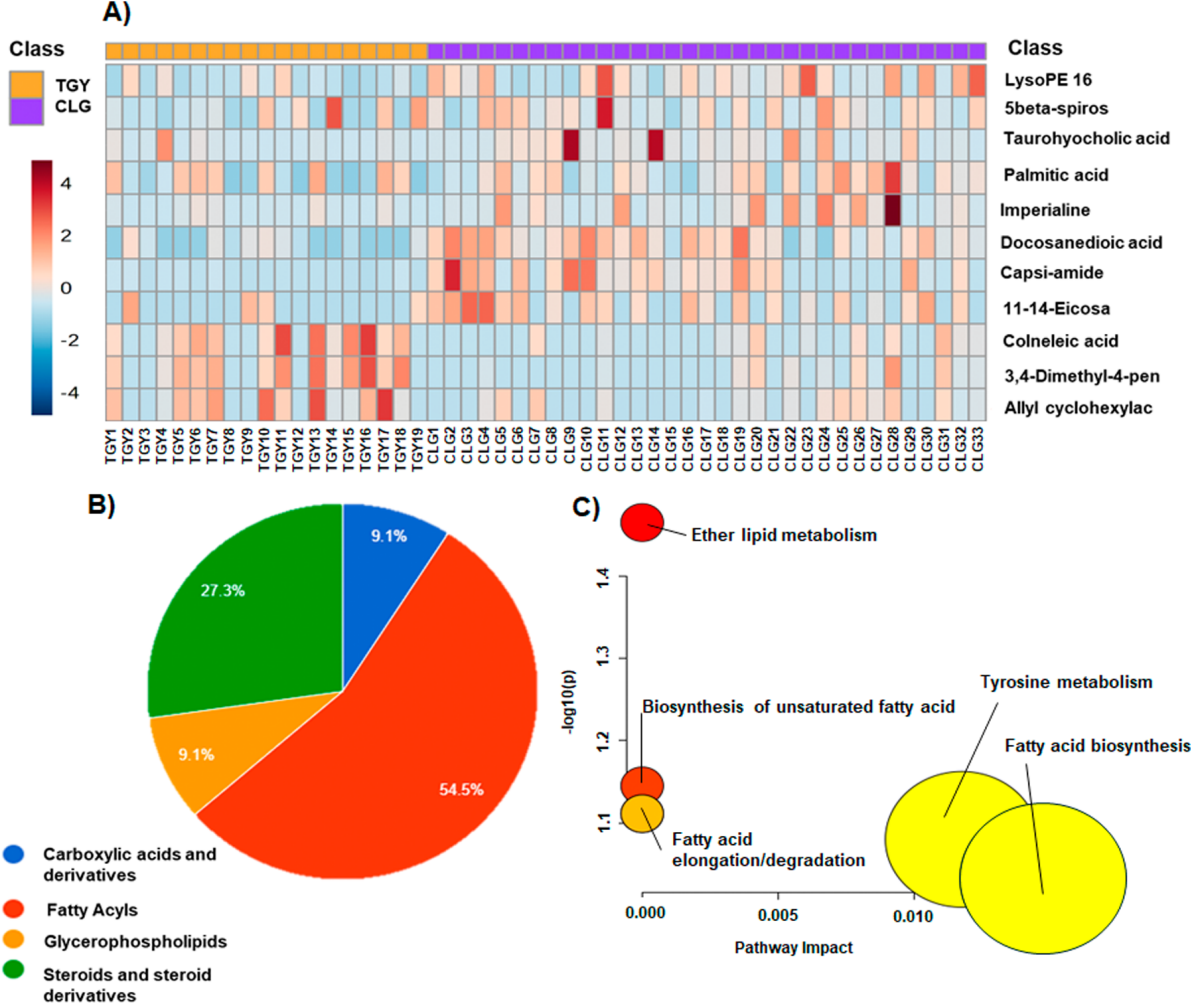
Comparison of fecal metabolomes between TYG and CLG groups. (A) Heatmap representing the relative abundance of significantly altered metabolites. (B) Main classes of metabolites that were altered between the groups. (C) Metabolic pathways affected by these alterations. TYG: yellow-legged gulls from La Tapa Saltworks; CLG: lesser black-backed from Cetina Saltworks.

The main altered class in the fecal metabolome comparison between TYG and CLG (Figure 3B) was fatty acyls (55%) followed by steroids and steroid derivatives (27%). The rest of the altered metabolites were carboxylic acids (9%) and glycerophospholipids (9%). Concretely, different changes in the levels of fatty acyls were observed. While we found significantly diminished levels of 3,4-dimethyl-5-pentyl-2-furanheptanoic acid, colneleic acid, and allyl cyclohexylacetate, the metabolites palmitic acid, 11,14-eicosadienoic acid, and docosanedioic acid increased in the CLG group compared to the TYG group. Additionally, the levels of steroids and steroid derivatives asparagoside A, imperialine, and taurohyocholic acid were decreased in TYG compared with LCG. Similarly, a diminution in the levels of capsiamide and LPE (16:0) was observed.

Considering the alteration of these metabolites, the analysis of the metabolic routes showed alteration in the ether lipid metabolism, the biosynthesis of unsaturated fatty acids, fatty acid elongation and degradation, tyrosine metabolism, and fatty acid biosynthesis (Figure 3C and Table S12).

Finally, Table S13 shows a summary of the main metabolic pathways altered for each comparison among the groups under study.

The chromatographic separation of metabolites in both ionization modes (Figure S4A,B) was optimized, and the 2D-PLS-DA showed a good grouping of samples in positive and negative ionization modes in all of the comparisons described above (Figure S5A–H). The PCA plots also demonstrated good clustering of the quality control samples (Figure S6A–H and Table S14). Furthermore, blanks were prepared by using the same procedure as the samples. They were analyzed at the beginning and end of the batch to assess injector contamination and the presence of artifacts in the UHPLC-QTOF-MS analysis (Figure S7A,B).

## Discussion

Our results suggest that spoonbill could be a good bioindicator of environmental pollution, as numerous differences were found in the fecal metabolome when comparing this waterbird in the OMBR and CBIBA, which have differential environmental quality as previously commented. Likewise, fatty acyls, bile acids, and sphingolipids were mainly altered in the fecal metabolome of OS vs CS. The main impacted metabolic routes are the fatty acid metabolism, the prime bile acid biosynthesis, and the sphingolipid metabolism. Many authors have reported fecal metabolic changes of these compounds in avian species according to the season, climate, or metal pollution.^20,23,25^ Given that samples from both areas were collected during the same season and shared similar climatic conditions, the primary difference is likely attributable to varying levels of contamination. In addition, contamination can indirectly affect the intestinal microbiota of waterfowl, either through the ingestion of contaminants or through changes in water and feed quality.^29^ The decrease in the levels of the most bile acids (BAs) in feces from OS compared to CS could be associated with microbiota disturbances due to contamination, since the microbiota plays a role in BA metabolism and any disruption could reduce their presence in feces.^30^ It is known that heavy metal exposure induces significant changes in the BA fraction of the fecal metabolome in mice^31^ and amphibians,^32^ although their levels in feces of waterfowl in contaminated areas have not been extensively studied. Despite that, variations in the composition of BAs have been observed in feces from chickens exposed to sources of pollution in the environment.^33^ Therefore, decreased levels of sphingolipids (SPLs) in OS vs CS could suggest alterations in the intestinal microbiota^34^ or liver damage (since these compounds are mainly synthesized in the liver) possibly caused by environmental pollution or low-quality diet.^35^ Additionally, diminution in SPLs could be also associated with the inhibition of the function of the enzymes responsible for producing these compounds caused by the oxidative stress induced by the exposure to heavy metals.^36,37^ Variations in the levels of SPLs have not been either widely studied in waterfowl exposed to contaminants, although significant changes in SPLs have been observed in chickens fed a mycotoxin-enriched diet.^38^ In this regard, studies in cell cultures have revealed the dysregulation of SPL metabolism due to heavy metal exposure.^39^

The altered classes of metabolites in the different waterfowl species within the same natural area (OS vs OG) were mainly FAs, GPLs, and BAs. The alteration could be associated with several factors such as migration or feeding patterns since, except for the spoonbill, which only feeds in natural habitats,^40^ the remaining species predominantly feed in natural and anthropogenic areas such as landfill sites.^18^ This reliance on natural habitats renders the spoonbill as a valuable bioindicator about the possible effect of environmental pollution in the protected area, offering a “snapshot” of the health state at a particular time point. In contrast, other waterfowl could serve as good bioindicators not only of the environmental quality of the natural area but also of the presence of anthropogenic sources close to the protected area that could provoke biological effects. Given the importance of FAs in inflammatory responses, thermoregulation, and cell membrane fluidity,^41^ the observed downregulation in the levels of FAs in black-headed gull (OG vs OS) is remarkable. This downregulation could also be related to pollution, which can have several effects on the digestive system of waterfowl, and in some cases, it can influence the absorption and excretion of FAs.^42^ In addition, contamination by heavy metals, pesticides, or other chemicals can affect the function of the liver and other organs related to fat metabolism,^43^ which could reduce the levels of FAs in feces. It is known that toxicants can produce oxidative stress in birds, causing upregulation of enzymes involved with FA synthesis.^44^ However, some studies have reported that the FA profiles depend on an interplay between diet, migratory strategy, and geographical barrier.^45^ Most of these variations seem to follow our predictions about habitat-related and seasonal differences in diet composition (although the change in diet covaried with ambient temperature). Moreover, Jensen et al. concluded that exposure to high heavy metal concentrations may alter the FA composition within birds, potentially reducing breeding changes and increasing mortality.^26^ On the other hand, the elevated levels of saturated fatty acids (SFAs) found in the feces of the OG against other waterfowl in the same area may be attributed to their ability to adapt to a variety of environments, including landfills, and to their diet, which includes insects, seeds, worms, debris, and carrion. In contrast, the decrease in SFAs observed in the OS vs OG could be the result of its diet, which consists more of natural sources such shrimp and fish rich in unsaturated and poor in saturated fatty acids.^46^ This dietary difference also supports the increase in unsaturated fatty acids identified in the feces of the OS vs OG.

The increase in the levels of GPLs found in the feces of OS may be attributed to the presence of heavy metals in the environment that increase oxidative stress in birds, causing alterations in the integrity of their cell membranes. As an adaptive mechanism to this damage, cells may increase the synthesis of GLPs, thus favoring the repair and maintenance of membrane structures. Several authors pointed out that the exposure to these pollutants is associated with an increase in the lipid peroxidation process, evidencing the alteration of membrane lipids and the activation of compensatory pathways to restore their functionality.^47^ In contrast, OG may exhibit lower excretion of these compounds due to a diet that is less rich in natural GPLs. The ability of black-headed gulls to adapt to different food sources allows them to thrive in a variety of environments, as they take advantage of organic debris found floating on the water or stranded on the shore. This makes it likely that black-headed gulls consume a higher proportion of processed foods low in natural GPLs.^48^

On the other hand, the observed increase in BAs in the feces of OG compared to OS; may stem from the presence of contaminants and processed foods in their feeding habitats, which can impact liver function and the downregulation of these compounds.^49^ Consequently, this may lead to reduced digestive efficiency, hindering the absorption of these compounds in the intestine and resulting in higher quantities being excreted in their feces.^50^ In contrast, the levels of BAs found in the feces of the OS were lower, as their feeding in natural environments promotes greater digestive efficiency, enabling a major complete absorption of BAs.

Comparing the fecal metabolomes of TYG vs TS, we also observed mainly a decrease in the levels of FAs and GPLs. These metabolic differences can be attributed to several differences, such as diet, habitat, and the mobility and bioaccumulation of contaminants.^51^ In this sense, the yellow-legged gull is omnivorous and opportunistic, and it feeds mainly on urban waste and food scraps in landfills.^52^ On the contrary, the stork is mainly carnivorous, although it can also feed on waste from landfills, does so less frequently than the yellow-legged gull, and its diet depends on a greater quantity of insects, amphibians, or small mammals.^53^ In summary, while the yellow-legged gull is more versatile and opportunistic in its feeding, the stork focuses more on its live prey in humid environments. Despite that, the migration patterns of these two waterfowls hold significant ecological importance, as they frequently undertake long journeys to seek food resources in landfills.^54^ Furthermore, due to its habitat, exposure to pollutants may vary. The yellow-legged gull prefers to live in coastal areas, ports, and beaches, indicating main exposure to marine pollution, but also approaches urban areas where it can take advantage of human waste.^53^ The storks prefer to live in marshes and wetlands, reflecting mostly the exposure to contamination from agricultural soils.^54^ Therefore, the reduction in GPLs may be attributed to diminished dietary intake of these compounds, owing to the greater tendency of the yellow-legged gull compared with the stork to consume contaminated food or human waste from landfills or coastal areas. The decrease in FAs could be linked to increased consumption of these compounds for the energy required for flight or thermoregulation in marine environments.^55,56^ Conversely, storks are becoming increasingly sedentary and may require less energy, leading to excretion of excess nutrients through feces.

Finally, we found mainly significant differences in the levels of FAs in TYG vs CLG. These two species belonging to the genus *Larus* are seabirds with similarities in appearance and size. However, they may present differences in diet, migration, or exposure to contaminants. Regarding diet, the yellow-legged gull prefers to feed on remains in landfills,^57^ and the lesser black-backed gull feeds mainly in rice fields and landfills, roosting in reservoirs, rivers, fishponds, and other waterbodies while also using marine habitats.^58,59^ Regarding migration, while yellow-legged gulls have a shorter migration period, more localized movements to nearby areas in winter,^60^ while lesser black-backed gulls migrate for longer distances, often crossing great distances from colder areas of Europe to southern Europe and Africa.^61^ Thus, yellow-legged gulls could be exposed to contaminants from anthropogenic waste.^62^ Differences in the levels of FAs have been reported in tissues from these two species, suggesting that the quality of the FA composition of yellow-legged gulls is lower than that in black–blacked gulls.^46^

Other studies in protected areas of southwest Spain focus on the use of the metabolome of *Procambarus clarkii* ^63–65^ and *Carcinus maenas* ^66^ as bioindicators of environmental pollution. However, studies using waterfowls are still lacking.

## Conclusions

This study provides new insights into the metabolism of different waterfowl species from different natural areas of southwestern Spain. The analysis by UHPLC-QTOF-MS allowed determining differences in the fecal metabolome that could be associated with environmental factors as well as migration or feeding patterns. We have associated the alteration of fecal metabolites in OS vs CS with the contamination of the natural areas, especially the OMBR that has been widely contaminated over the years. Moreover, migration and feeding patterns have been associated with fecal metabolome differences of OS vs OG and TYG vs TS in the same natural area, as well as the differences in the fecal metabolome of TYG vs CLG. In conclusion, the area where fecal samples were collected, along with feeding habits, migration patterns, and metabolism, significantly influences the fecal metabolome of each waterfowl species. This fact emphasizes their importance as bioindicators of environmental health. Consequently, monitoring these bioindicator waterbirds is crucial for assessing environmental quality and delving into the potential biological effects of pollutants.

## Supplementary Material



## Notes

Collected fecal samples from different waterfowls across the study areas (Table S1), the Batch Recursive Feature Extraction parameters used for the UHPLC-QTOF-MS analysis (Table S2), *Q* ^2^ and *R* ^2^ values obtained from all PLS-DA comparisons (Table S3), altered fecal metabolites ordered by class for the comparison between spoonbills from Odiel Marshes (OS) and Cetina Saltworks (CS) (Table S4), MS/MS fragments used for their annotation (Table S5), pathway analysis results for these altered metabolites (Table S6), altered fecal metabolites ordered by class when comparing spoonbills from Odiel Marshes (OS) and black-headed gull in the Odiel marshes (OG), as well as yellow-legged gull from Tapa saltworks (TYG) and storks from La Tapa Saltworks (TS) (Table S7), corresponding MS/MS fragments used for annotation (Table S8), the pathway analysis for altered metabolites in OS, OG, TYG, and TS groups (Table S9), altered metabolites ordered by subclass from the comparison between yellow-legged gulls (TYG) and lesser black-backed gulls (CLG) (Table S10), the MS/MS fragments used for their annotation (Table S11), pathway analysis results for the same comparison (Table S12), main altered metabolic pathways identified across all comparisons (Table S13), the coefficient of variation (CV) of gut metabolites calculated in quality control (QC) samples (Table S14), the location of the study zones (Figure S1), the main metabolite classes altered between groups (Figure S2), metabolic pathways affected by these changes (Figure S3), a typical fecal metabolome profile obtained by UHPLC-QTOF-MS in both ionization modes (Figure S4), the PLS-DA plots showing group separations (Figure S5), the PCA plots demonstrating QC clustering (Figure S6), blank sample profiles obtained in positive and negative ionization (Figure S7) (PDF)
