# Supplementary material for: Environmental Quality Assessment Using Fecal Metabolomics in Waterfowl from Protected Wetlands in Southwest Spain
Source: ACS ES T Water. 2026 Feb 10;6(3):1775–85. doi: 10.1021/acsestwater.5c01312 (PMC12994005; doi:10.1021/acsestwater.5c01312)

Environmental quality assessment using fecal metabolomics in waterfowl from protected wetlands in Southwest Spain

Cristina Navarro Fernández [a £], *Belén Callejón-Leblic* [a £], *Ángel Rafael Domínguez-Bustos*[a], *Isabel Molina*[b] *Francisco Hortas*[c*], *Tamara García-Barrera*[a*]

[a]Research Center for Natural Resources Health and the Environment (RENSMA). Department of Chemistry, Faculty of Experimental Sciences, University of Huelva, Campus El Carmen, Fuerzas Armadas Ave., 21007, Huelva, Spain.

[b]Andalusian Network of Centres for the Recovery of Threatened Species. CREA Dunas de San Anton. Camino de los Enamorados s/n. El Puerto de Santa Maria. Spain.[c]Department of Biology, Institute of Marine Research (INMAR). University of Cadiz, 11510, Puerto Real, Spain. *tamara@dqcm.uhu.es; *francisco.hortas@gm.uca.es

[£]Both authors contributed equally

Table of contents

Table S1	Collected fecal samples from different avian species per area
Table S2	Batch Recursive Feature Extraction parameters (UHPLC-QTOF-MS analysis)
Table S3	Table S3. Q^2 and R^2 values from all the PLS-DA comparisons in the study. Common spoonbills (OS), Black-headed Gull (OG group) from Odiel Marshes, yellow-legged gulls (TYG) and storks (TS) from La Tapa Saltworks and lesser black-backed from Cetina Saltsworks (CLG).
Table S4	Fecal altered metabolites ordered by class from comparing OS and CS groups. RT: retention time, FC: Fold change, IM: Ionization mode, OS: Spoonbills from Odiel Marshes, CS: spoonbill from Cetina Saltworks, lysophosphatidylethanolamine (LPE), phosphatidylcholine (PC); ceramide (Cer); *p*-value obtained from one-way ANOVA followed by Tuckey Test and corrected by Benjamini-Hochberg multiple post-correction.
Table S5	List of fragments of the MS/MS experiment for the annotation of altered metabolites in the comparison of OS and CS groups. OS: Spoonbills from Odiel Marshes, CS: spoonbill from Cetina Saltworks, lysophosphatidylethanolamine (LPE), phosphatidylcholine (PC); ceramide (Cer).
Table S6	Pathway analysis details of altered metabolites in OS and CS groups. Match Status: the number of altered metabolites of the total metabolites involved in the route, with the p-value calculated from the enrichment analysis; Impact: pathway impact value calculated from pathway topology analysis.
Table S7	Fecal altered metabolites ordered by class from comparing OS and OG groups, and TYG and TS. RT: retention time, FC: Fold change, IM: Ionization mode, OS: Spoonbills from Odiel Marshes, OG: black-headed gulls from Odiel Marshes, TYG: yellow-legged gulls and TS: storks from La Tapa Saltworks. Lysophosphatidylethanolamine (LPE), phosphatidylcholine (PC); ceramide (Cer); *p*-value obtained from one-way ANOVA followed by Tuckey Test and

	corrected by Benjamini-Hochberg multiple post-correction.
Table S8	List of fragments of the MS/MS experiment for the annotation of altered metabolites in comparing OS and OG groups, and TYG and TS. OS: Spoonbills from Odiel Marshes, OG: black-headed gulls from Odiel Marshes, TYG: yellow-legged gulls, and TS: storks from the La Tapa Saltworks, lysophosphatidylethanolamine (LPE), phosphatidylcholine (PC); ceramide (Cer).
Table S9	Pathway analysis details of altered metabolites in OS: Spoonbills from Odiel Marshes, OG: black-headed gulls from Odiel Marshes, TYG: yellow-legged gulls and TS: storks from La Tapa Saltworks. Match Status: the number of altered metabolites of the total metabolites involved in the route, with the p-value calculated from the enrichment analysis; Impact: pathway impact value calculated from pathway topology analysis.
Table S10	Fecal altered metabolites ordered by subclass from comparing TYG and CLG groups. RT: retention time, FC: Fold change, IM: Ionization mode, TYG: yellow-legged gulls from La Tapa Saltworks and CLG: lesser black-backed from Cetina Saltsworks. Lysophosphatidylethanolamine (LPE); *p*-value obtained from one-way ANOVA followed by Tuckey Test and corrected by Benjamini-Hochberg multiple post-correction.
Table S11	List of fragments of the MS/MS experiment for the annotation of altered metabolites in the comparison of TYG and CLG groups. TYG: yellow-legged gulls from La Tapa Saltworks and (CLG) lesser black-backed from Cetina Saltsworks. Lysophosphatidylethanolamine (LPE).
Table S12	Pathway analysis details of altered metabolites in TYG: yellow-legged gulls from La Tapa Saltworks and (CLG) lesser black-backed from Cetina Saltsworks. Match Status: the number of altered metabolites of the total metabolites involved in the route, with the p-value calculated from the enrichment analysis; Impact: pathway impact value calculated from pathway topology analysis.

Table S13	Summary of the main altered metabolic pathways for each comparison.
Table S14	Coefficient of variation (CV) of gut metabolites calculated in quality control (QC) samples. Lysophosphatidylcholine (LPC), lysophosphatidylethanolamine (LPE), phosphatidylethanolamine (PE); phosphatidylglycerol (PG); phosphatidylserine (PS); monoglyceride (MG).
Figure S1	Location of the study zones.
Figure S2	The figure highlights the main classes of metabolites that were altered between the groups of A) OS and OG groups and B) TS and TYG groups.
Figure S3	The figure illustrates the metabolic pathways affected by these alterations in A) OS and OG groups and B) TS and TYG groups.
Figure S4	Typical fecal metabolome profiling of waterfowl using UHPLC-QTOF-MS analysis in A) positive and B) negative ionization modes.
Figure S5	The PLS-DA plots demonstrated good separation between groups for OS and CS groups in A) positive mode and B) negative modes; OS vs OG groups in C) positive mode and D) negative modes; TYG and TS groups in E) positive mode and F) negative modes; and TYG and CLG groups in G) positive mode and H) negative modes.
Figure S6	The PCA plots demonstrated good clustering of the quality control samples for OS and CS groups in A) positive mode and B) negative modes; OS vs OG groups in C) positive mode and D) negative modes; TYG and TS groups in E) positive mode and F) negative modes; and TYG and CLG groups in G) positive mode and H) negative modes.
Figure S7	Blank samples from (A) UHLC-ESI(+)-QTOF-MS (B) UHPLC-ESI(-)-QTOF-MS.

Table S1. Collected fecal samples from different avian species per area

Specie	**Area**	**City**	**n**
Chroicocephalus ridibundus	Odiel Marshes	Huelva	34
Larus michahellis	La Tapa Saltworks	Cádiz	19
Ciconia ciconia	La Tapa Saltworks	Cádiz	42
Platalea leucorodia	Odiel Marshes	Huelva	10
Platalea leucorodia	Cetina Saltworks	Cádiz	38
Larus fuscus	Cetina Saltworks	Cádiz	33

Table S2. Batch Recursive Feature Extraction parameters (UHPLC-QTOF-MS analysis)

**Batch Recursive Feature Extraction**	
**Extraction**	
Peak weight	> 5000 counts
**Ion species**	
Positive Mode	$[M+H]^+$, $[M+Na]^+$
Negative Mode	$[M-H]^-$, $[M-Cl]^-$
**Integration**	Agile 2
**Alignment Parameters**	
RT Tolerance	0.00% ± 0.3 min
Mass Tolerance	10 ppm ± 2.00 mDa
**Molecular Feature Extraction Filters**	
Score (MFE)	> 90
**Tolerance and EIC**	
Masses	± 10.00 ppm
RT	± 0.300 min
Possible m/z	Symmetric (ppm)
**Peak Filter**	
Absolute area	> 5000 counts
**Chromatogram Format**	**Centroid**
**Peak Spectrum**	
Average scans	10 % of peak height
**Find by ion filters**	
Score (Tgt)	> 95
**Entities**	
The number of entities found (OG vs CG)	Positive Mode: 235
	Negative Mode: 853
The number of entities found (OG vs OS / TYG vs TS)	Positive Mode: 225
	Negative Mode: 395
The number of entities found (TYG vs CLG)	Positive Mode: 213
	Negative Mode: 584
Number of significant entities found (OG vs CG)	Positive Mode: 129
	Negative Mode: 277
Number of significant entities found (OG vs OS / TYG vs TS)	Positive Mode: 214
	Negative Mode: 644
Number of significant entities found (TYG vs CLG)	Positive Mode: 131
	Negative Mode: 197

Table S3. Q^2 and R^2 values from all the PLS-DA comparisons in the study. Eurasian spoonbills (OS), Black-headed gulls (OG group) from Odiel Marshes, yellow-legged gulls (TYG) and storks (TS) from La Tapa Saltworks and lesser black-backed from Cetina Saltsworks (CLG).

**Parameters**	**Q^2**				**R^2Y**			
**Groups**	**OS vs CS**	**OS vs OG**	**TYG vs TS**	**TYG vs CLG**	**OS vs CS**	**OS vs OG**	**TYG vs TS**	**TYG vs CLG**
**ESI(+)-UHPLC-QTOF-MS**	0.499	0.935	0.235	0.261	0.879	0.972	0.538	0.418
**ESI(-)-UHPLC-QTOF-MS**	0.774	0.945	0.226	0.784	0.945	0.979	0.836	0.871

Table S4. Fecal altered metabolites ordered by class from comparing OS and CS groups. RT: retention time, FC: Fold change, IM: Ionization mode, OS: Spoonbills from Odiel Marshes, CS: spoonbill from Cetina Saltworks, lysophosphatidylethanolamine (LPE), phosphatidylcholine (PC); ceramide (Cer); *p*-value obtained from one-way ANOVA followed by Tuckey Test and corrected by Benjamini-Hochberg multiple post-correction

Experimental mass (Da)	Theoretical mass (Da)	RT (min)	Compounds	[OS] vs [CS]		IM	Adduct	Class	Subclass	Score (DB)
				FC	p value					
326.1914	326.1915	13.52	2-Dodecylbenzenesulfonic acid	0.21	0.0001	ESI-	$[M-H]^-$	Benzene and substituted derivatives	Benzenesulfonic acids and derivatives	97.31
312.176	312.1759	12.78	N-Undecylbenzenesulfonic acid	0.10	0.02	ESI-	$[M-H]^-$	Benzene and substituted derivatives	Benzenesulfonic acids and derivatives	98.69
228.146	228.14739	0.56	L-isoleucyl-L-proline	1.97	0.0001	ESI+	$[M+H]^+$	Carboxylic acids and derivatives	Amino acids, peptides, and analogues	93.95
244.2036	244.2038	10.72	2-hydroxy myristic acid	0.41	0.0005	ESI-	$[M-H]^-$	Fatty Acyls	Fatty acids and conjugates	99.74
300.2659	300.2664	11.23	10-hydroxystearic acid	0.07	0.0001	ESI-	$[M-H]^-$	Fatty Acyls	Fatty acids and conjugates	96.63
272.2348	272.2351	11.57	16-Hydroxy hexadecanoic acid	0.21	0.0001	ESI-	$[M-H]^-$	Fatty Acyls	Fatty acids and conjugates	98.43
256.24	256.2402	13.33	Palmitic Acid	0.42	0.0001	ESI-	[M-H]-	Fatty Acyls	Fatty acids and conjugates	97.3
784.5851	783.5778	9.16	PC(22:2/14:1)	0.40	0.0001	ESI+	$[M+H]^+$	Glycerophospholipids	Glycerophosphocholines	97.51
509.3468	509.3481	11.40	LPE(20:0)	2.06	0.01	ESI-	$[M-H]^-$	Glycerophospholipids	Glycerophosphoethanolamines	97.36
477.2545	477.2548	8.97	Epothilone C	5.25	0.0001	ESI-	$[M-H]^-$	Macrolides and analogues	Epothilones and analogues	95.74
509.4806	509.4807	17.29	Cer(d18:1/14:0)	0.37	0.0001	ESI+	$[M+H]^+$	Sphingolipids	Ceramides	92.55
481.4512	481.4494	16.35	Cer(d18:1/12:0)	0.05	0.0001	ESI-	$[M+H]^+$	Sphingolipids	Ceramides	97.61
481.4475	433.3192	7.31	Lithocholic acid glycine conjugate	0.04	0.0001	ESI-	$[M-H]^-$	Steroids and steroid derivatives	Bile acids, alcohols and derivatives	95.76
392.2924	392.2926	10.84	3,7-Dihydroxy-5-cholanoic acid	0.11	0.0001	ESI-	$[M-H]^-$	Steroids and steroid derivatives	Bile acids, alcohols and derivatives	99.66
532.3056	532.3069	8.94	5-Cyprinol sulfate	0.16	0.0001	ESI-	$[M-H]^-$	Steroids and steroid derivatives	Bile acids, alcohols and derivatives	95.35
499.2961	499.2967	6.42	Tauroursodeoxycholic acid	5.44	0.0001	ESI-	$[M-H]^-$	Steroids and steroid derivatives	Bile acids, alcohols and derivatives	99.03
408.288	408.2875	7.96	3,6,7-Trihydroxy-5-cholanoic acid	0.18	0.0001	ESI-	$[M-H]^-$	Steroids and steroid derivatives	Bile acids, alcohols and derivatives	96.46
515.2914	515.2916	8.45	Taurohyocholic acid	2.87	0.0004	ESI-	$[M-H]^-$	Steroids and steroid derivatives	Bile acids, alcohols and derivatives	96.13
316.2402	316.2402	12.64	5-alpha-pregnan-3,20-dione	0.03	0.0001	ESI-	$[M-H]^-$	Steroids and steroid derivatives	Pregnane steroids	97.47

Table S5. List of fragments of the MS/MS experiment for the annotation of altered metabolites in the comparison of OS and CS groups. OS: Spoonbill from Odiel Marshes, CS: spoonbill from Cetina Saltworks, lysophosphatidylethanolamine (LPE), phosphatidylcholine (PC); ceramide (Cer).

Compound	Exp. Mass (m/z)	Fragmentos (m/z)
2-Dodecylbenzenesulfonic acid	326.1914	43.0547<br>82.9802<br>106.9802<br>127.1487<br>245.2269<br>307.1732<br>325.1837
N-Undecylbenzenesulfonic acid	312.176	43.0547<br>80.9646<br>104.9646<br>113.1330<br>199.0429<br>283.1368<br>311.1681
L-isoleucyl-L-proline	228.146	57.0698<br>69.0698<br>70.0651<br>86.0964<br>98.0600<br>142.0499<br>229.1547
2-hydroxy myristic acid	244.2036	72.9931<br>169.1961<br>181.1961<br>197.1910<br>199.2067<br>225.1860<br>243.1965
10-hydroxystearic acid	300.2659	99.1173<br>111.1173<br>113.1330<br>125.1330<br>141.1643<br>253.2531<br>299.2586
16-Hydroxy hexadecanoic acid	272.2348	27.0234<br>29.0027<br>31.0183<br>41.0027<br>44.9977<br>207.2113<br>235.2062
Palmitic Acid	256.24	59.0133<br>71.0860<br>85.1017<br>99.1173<br>113.1330<br>195.2112<br>237.2218
PC(22:2/14:1)	784.5851	576.4029<br>448.2827<br>558.3924<br>184.0738<br>86.0969<br>125.0003<br>104.1075
LPE(20:0)	509.3468	122.9852<br>137.0009<br>140.0118

		152.9958<br>182.0223<br>196.0380<br>214.0485
Cer(d18:1/14:0)	509.4806	510.4886<br>492.4781<br>474.4675<br>462.4675<br>282.2797<br>264.2691
Cer(d18:1/12:0)	481.4512	482.4573<br>464.4467<br>446.4361<br>434.4361<br>282.2796<br>264.2691
Lithocholic acid glycine conjugate	481.4475	74.0242<br>76.0398<br>98.0242<br>100.0034<br>102.0191<br>317.2844<br>402.3008
3,7-Dihydroxy-5-cholanoic acid	392.2924	59.0138<br>329.2849<br>347.2955<br>355.2642<br>373.2748<br>391.2853
5-Cyprinol sulfate	532.3056	417.3374<br>419.3166<br>421.3323<br>433.3323<br>483.2785<br>485.2942<br>495.2785
Tauroursodeoxycholic acid	499.2961	126.0225<br>149.9861<br>152.0018<br>166.0174<br>329.2844<br>347.2950<br>357.2794
3,6,7-Trihydroxy-5b-cholanoic acid	408.288	59.0139<br>345.2799<br>371.2592<br>363.2905<br>389.2697<br>407.2803
Taurohyocholic acid	515.2914	166.0174<br>345.2794<br>363.2899<br>371.2586<br>373.2743<br>388.2852<br>389.2692
5-alpha-pregnan-3,20-dione	316.2402	41.0033<br>287.2380<br>273.2224<br>297.2224<br>299.2017<br>315.2330

Table S6. Pathway analysis details of altered metabolites in OS and CS groups. Match Status: the number of altered metabolites of the total metabolites involved in the route, with the p-value calculated from the enrichment analysis; Impact: pathway impact value calculated from pathway topology analysis.

**Pathway Name**	**Match Status**	**p**	**Impact**
Taurine and hypotaurine metabolism	1/8	0.016216	0.0
Sphingolipid metabolism	1/32	0.06381	0.21576
Biosynthesis of unsaturated fatty acids	1/36	0.07159	0.0
Fatty acid elongation	1/39	0.077396	0.0
Fatty acid degradation	1/39	0.077396	0.0
Primary bile acid biosynthesis	1/46	0.090851	0.03799
Fatty acid biosynthesis	1/47	0.092762	0.01473

Table S7. Fecal altered metabolites ordered by class from comparing OS and OG groups, and TYG and TS. RT: retention time, FC: Fold change, IM: Ionization mode, OS: Spoonbills from Odiel Marshes, OG: Black-headed gulls from Odiel Marshes, TYG: yellow-legged gulls and TS: storks from La Tapa Saltworks. lysophosphatidylethanolamine (LPE), phosphatidylcholine (PC); ceramide (Cer); *p*-value obtained from one-way ANOVA followed by Tuckey Test and corrected by Benjamini-Hochberg multiple post-correction

Experimental Mass (Da)	Theorethical Mass (Da)	RT (min)	Compounds	[OS] vs [OG]		[TYG] vs [TS]		IM	Adduct	Subclass	Score (DB)
				FC	p value	FC	p value				
286.0951	286.0953	0.87	3',4'-Dihydrodiol	-	-	3.66	0.009	ESI+	$[M+H]^+$	Imidazolidines	97.45
326.1914	326.1915	12.78	2-Dodecylbenzenesulfonic acid	-	-	0.28	0.005	ESI-	$[M-H]^-$	Benzenesulfonic acids and derivatives	97.32
312.1758	312.1759	14.32	N-Undecylbenzenesulfonic acid	-	-	0.38	0.0001	ESI-	$[M-H]^-$	Benzenesulfonic acids and derivatives	97.17
378.1615	378.1613	2.13	Zinnolide	2.07	0.005	1.26	0.01	ESI+	$[M+H]^+$	Phenethylamines	99.28
492.2731	492.2648	9.28	Mucronine B	-	-	0.72	0.0001	ESI-	$[M-H]^-$	$[M-H]^-$	97.11
216.0121	216.111	6.19	N2,N5-Dibenzoyl-L-ornithine	-	-	0.000001	0.0001	ESI-	$[M-H]^-$	Amino acids, peptides, and analogues	96.56
269.2811	269.2718	12.70	Capsiamide	0.72	0.0003	-	-	ESI+	$[M+H]^+$	Carboxylic acid derivatives	93.83
292.0912	292.0906	0.49	Edetate	0.34	0.0001	-	-	ESI+	$[M+H]^+$	Tetracarboxylic acids and derivatives	95.72
204.0214	204.1184	9.39	Hexyl 3-mercaptobutanoate	1.01	0.0001	-	-	ESI-	$[M-H]^-$	Fatty acid esters	95.5
368.293	368.2926	13.51	Octadecyl fumarate	-	-	0.14	0.0001	ESI-	$[M-H]^-$	Fatty acid esters	96.63
330.2556	330.2558	12.85	Eicosapentaenoic Acid ethyl ester	0.03	0.0001	0.15	0.0001	ESI-	$[M-H]^-$	Fatty acid esters	98.53
300.2659	300.2664	13.22	10-hydroxystearic acid	0.09	0.0001	0.40	0.02	ESI-	$[M-H]^-$	Fatty acids and conjugates	97.67
294.2174	294.2194	10.59	3,4-Dimethyl-5-pentyl-2-furanheptanoic acid	4.01	0.0009	-	-	ESI-	$[M-H]^-$	Fatty acids and conjugates	96.25
294.2185	294.2194	10.49	Colneleic acid	7.94	0.0001	0.36	0.005	ESI-	$[M-H]^-$	Fatty acids and conjugates	95.86
280.2401	284.2715	12.78	Stearolic acid	2.68	0.01	0.32	0.0001	ESI-	$[M-H]^-$	Fatty acids and conjugates	96.85
340.2599	340.2019	13.49	Sterculic acid	-	-	0.25	0.001	ESI-	$[M-H]^-$	Fatty acids and conjugates	95.75
254.2014	254.2245	12.45	9-Hexadecenoate	6.91	0.02	0.16	0.0002	ESI-	$[M-H]^-$	Fatty acids and conjugates	97.09
370.3088	370.3083	14.60	Docosanedioic acid	-	-	0.86	0.0001	ESI+	$[M+H]^+$	Fatty acids and conjugates	98.64
272.2352	272.2351	12.02	16-Hydroxy hexadecanoic acid	0.20	0.0001	-	-	ESI-	$[M-H]^-$	Fatty acids and conjugates	97.46
256.2404	256.2402	13.33	Palmitic Acid	-	-	0.46	0.0001	ESI-	$[M-H]^-$	Fatty acids and conjugates	98.39

356.1228	356.3290	15.23	22-Hydroxydocosanoic acid	0.11	0.0001	-	-	ESI-	$[M-H]^-$	Fatty acids and conjugates	99.47
354.3132	353.3061	14.34	22-Oxo-docosanoate	0.02	0.0001	0.35	0.0001	ESI-	$[M-H]^-$	Fatty acids and conjugates	97.38
316.1763	316.2613	11.11	9,10-Dihydroxystearic acid	5.33	0.0001	-	-	ESI-	$[M-H]^-$	Fatty acids and conjugates	97.95
255.2149	255.2562	12.32	Palmitic amide	-	-	0.88	0.0007	ESI+	$[M+H]^+$	Fatty amides	94.62
296.1375	296.2351	11.08	12-Hydroxy-8,10-octadecadienoic acid	1.56	0.04	-	-	ESI-	$[M-H]^-$	Lineolic acids and derivatives	96.25
574.4591	574.4597	13.81	DG(15:0/18:4)	-	-	0.25	0.0003	ESI-	$[M-H]^-$	Lineolic acids and derivatives	95.88
438.2749	438.2746	13.53	LysoPE(18:0)	-	-	0.34	0.0001	ESI-	$[M-H]^-$	Glycerophosphates	95.25
694.4774	648.473	13.52	PA(16:0/16:0)	-	-	0.91	0.0001	ESI-	$[M-H]^-$	Glycerophosphates	96.56
523.2911	523.3637	11.61	LysoPC(18:0)	-	-	0.14	0.0001	ESI+	$[M+H]^+$	Glycerophosphocholines	91.1
784.5850	783.5778	9.16	PC(22:2/14:1)	1.24	0.0001	-	-	ESI+	$[M+H]^+$	Glycerophosphocholines	98.31
477.2849	477.2855	10.40	LysoPE(18:2)	3.25	0.0001	-	-	ESI-	$[M-H]^-$	Glycerophosphoethanolamines	97.47
509.3481	509.3481	12.74	LysoPE(20:0)	0.83	0.0001	-	-	ESI-	$[M-H]^-$	Glycerophosphoethanolamines	98.27
507.3317	507.3324	10.59	LysoPE(20:1)	-	-	0.96	0.02	ESI-	$[M-H]^-$	Glycerophosphoethanolamines	97.66
481.3163	481.3168	11.27	LysoPE(18:0)	3.63	0.02	0.45	0.0001	ESI+	$[M-H]^-$	Glycerophosphoethanolamines	91.66
477.2543	477.2548	8.98	Epothilone C	0.02	0.0001	-	-	ESI-	$[M-H]^-$	Epothilones and analogues	95.74
475.1306	475.3005	4.38	Netilmicin	1.23	0.0001	-	-	ESI+	$[M+H]^+$	Carbohydrates and carbohydrate conjugates	94.17
582.3144	582.4342	11.54	Polidocanol	1.55	0.0001	0.55	0.003	ESI+	$[M+H]^+$	Ethers	98.24
328.0778	328.2402	12.53	2-Methyl-5-(8,11,14-pentadecatrienyl)-1,3-benzenediol	0.33	0.0001	0.75	0.0001	ESI+	$[M+H]^+$	Benzenediols	93.89
304.2399	300.2089	12.67	Dihydroabietic acid	0.41	0.0001	0.40	0.0004	ESI-	$[M-H]^-$	Diterpenoids	98.17
330.0264	330.2558	12.03	Ethyl abietic acid	-	-	0.29	0.0001	ESI-	$[M-H]^-$	Diterpenoids	97.29
328.2904	328.2977	14.27	2-hydroxyphytanic acid	0.08	0.0001	-	-	ESI-	$[M-H]^-$	Diterpenoids	98.36
234.1616	234.1619	10.13	Saussurea lactone	0.76	0.0001	1.38	0.02	ESI-	$[M-H]^-$	Diterpenoids	99.53
228.1102	228.1184	9.39	Menthone 8-thioacetate	1.39	0.0001			ESI-	$[M-H]^-$	Monoterpenoids	98.48
310.1114	310.2144	9.72	Auxin b	0.86	0.0001			ESI-	$[M-H]^-$	Monoterpenoids	97.09
424.3378	424.3341	13.55	alpha-Tocotrienol	-	-	0.32	0.005	ESI+	$[M+H]^+$	Quinone and hydroquinone lipids	93.01
632.3911	632.3924	9.74	Cloversaponin I	-	-	0.52	0.0001	ESI-	[M-H]-	Terpene glycosides	98.9
596.3856	596.3865	13.85	Astaxanthin	0.12	0.0001	-	-	ESI+	$[M+H]^+$	Tetraterpenoids	95.2
268.0809	268.0807	0.61	Inosine	5.80	0.0001	-	-	ESI-	$[M-H]^-$	Purine nucleosides	95.05

565.5401	565.5433	10.18	Cer(d18:0/18:1)	0.90	0.0001	-	-	ESI-	$[M-H]^-$	Ceramides	96.16
392.2753	392.2926	8.12	3,7-Dihydroxy-5-cholan-24-Oic acid	0.09	0.0001	-	-	ESI-	$[M-H]^-$	Bile acids, alcohols and derivatives	99.83
465.3081	465.309	7.28	Glycocholic acid	2.51	0.0001	-	-	ESI-	$[M-H]^-$	Bile acids, alcohols and derivatives	97.1
515.2915	515.2916	8.45	Taurohyocholic acid	0.43	0.009	0.66	0.0001	ESI-	$[M-H]^-$	Bile acids, alcohols and derivatives	95.67
499.2951	499.2967	6.43	Tauroursodeoxycholic acid	0.04	0.002	5.95	0.0001	ESI-	$[M-H]^-$	Bile acids, alcohols and derivatives	95.93
433.2999	433.3192	9.42	Lithocholic acid taurine conjugate	0.01	0.0001	-	-	ESI-	$[M-H]^-$	Bile acids, alcohols and derivatives	98.16
696.5532	696.5845	9.16	Cholesteryl docosahexaenoic acid	0.11	0.002	-	-	ESI+	$[M+H]^+$	Steroid esters	95.37
429.3247	429.3242	13.44	Imperialine	-	-	1.04	0.0001	ESI+	$[M+H]^+$	Steroidal alkaloids	97.79

Table S8. List of fragments of the MS/MS experiment for the annotation of altered metabolites in comparing OS and OG groups, and TYG and TS. OS: Spoonbills from Odiel Marshes, OG: Black-headed gulls from Odiel Marshes, TYG: yellow-legged gulls, and TS: storks from the La Tapa Saltworks, lysophosphatidylethanolamine (LPE), phosphatidylcholine (PC); ceramide (Cer).

Compounds	Exp. Mass (m/z)	Fragmentos (m/z)
3,4-Dihydrodiol	289.1011	160.1126<br>170.0606<br>172.0762<br>184.0762<br>186.0919<br>197.0603<br>198.0919
2-Dodecylbenzenesulfonic acid	326.1914	43.0547<br>82.9802<br>106.9802<br>127.1487<br>245.2269<br>307.1732<br>325.1837
N-Undecylbenzenesulfonic acid	312.1758	43.0547<br>80.9646<br>104.9646<br>113.1330<br>199.0429<br>283.1368<br>311.1681
Mucronine B	492.2731	107.0855<br>112.0757<br>148.1121<br>176.1070<br>178.1226<br>216.1383<br>261.1961
Capsiamide	269.2811	40.0193<br>58.0298<br>197.2275<br>224.2384<br>226.2540<br>250.2540<br>268.2646
Edetate	292.0912	116.0712<br>130.0140<br>132.0297<br>134.0453<br>142.0504<br>156.0297<br>158.0453
Hexyl 3-mercaptobutanoate	204.0214	55.0548<br>57.0704<br>69.0704<br>83.0861<br>87.0446<br>153.1279<br>205.1262
Octadecyl fumarate	368.293	71.0861<br>80.9977<br>99.0082

		113.1330<br>127.1487<br>139.1487<br>141.1643
Eicosapentaenoic Acid ethyl ester	330.2556	123.1174<br>139.0759<br>141.0916<br>161.1330<br>163.1487<br>181.1229<br>201.1643<br>203.1800
10-hydroxystearic acid	300.2659	157.1592<br>169.1229<br>183.1385<br>185.1178<br>187.1334<br>201.1491<br>199.1334
3,4-Dimethyl-5-pentyl-2-furanheptanoic acid	294.2174	179.1436<br>183.1749<br>191.1436<br>193.1592<br>219.1385<br>221.1178<br>223.1334
Colneleic acid	294.2185	137.0966<br>139.0759<br>139.1123<br>141.0916<br>143.1072<br>145.1229<br>151.0759
Stearolic acid	280.2401	127.1487<br>139.1487<br>141.1643<br>153.1643<br>155.1800<br>167.1800<br>169.1956
Sterculic acid	340.2599	207.2118<br>219.2118<br>221.2275<br>231.2118<br>233.2275<br>245.2275<br>247.2431
9-Hexadecenoate	254.2014	85.0290<br>87.0446<br>97.1017<br>113.0603<br>193.1956<br>195.2113<br>205.1956
Docosanedioic acid	370.3088	129.0916<br>185.1542<br>199.1698<br>213.1855<br>227.2011<br>241.2168

		255.2324
16-Hydroxy hexadecanoic acid	272.2352	45.0340<br>55.0184<br>59.0133<br>167.1436<br>181.1592<br>181.1956<br>193.1956
Palmitic Acid	256.2404	59.0133<br>71.0860<br>85.1017<br>99.1173<br>113.1330<br>195.2112<br>237.2218
22-Hydroxydocosanoic acid	356.1228	89.0239<br>265.2895<br>267.3052<br>277.2895<br>279.3052<br>281.2844<br>283.3001
22-Oxo-docosanoate	354.3132	281.3214<br>289.2901<br>291.3057<br>293.2850<br>305.2850<br>307.2643<br>307.3006
9,10-Dihydroxystearic acid	316.1763	127.1123<br>129.1279<br>139.1123<br>141.1279<br>143.1072<br>143.1436<br>153.1279
Palmitic amide	255.2149	71.0861<br>85.1017<br>113.1330<br>127.1487<br>141.1643<br>155.1800<br>169.1956
12-Hydroxy-8,10-octadecadienoic acid	296.1375	181.1229<br>193.1229<br>209.1178<br>211.1334<br>223.1334<br>225.1491<br>231.2113
DG(15:0/18:4)	574.4591	592.4941<br>575.4676<br>557.4570<br>333.2430<br>299.2586
LPE(18:0)	438.2749	140.0118<br>152.9958<br>182.0224<br>196.0380<br>214.0486

		239.2744<br>265.2537
PA(16:0/16:0)	694.4774	78.9591<br>96.9696<br>211.2431<br>237.2224<br>255.2330<br>391.2255<br>409.2361
PC(22:2/14:1)	784.5850	317.2850<br>321.3163<br>333.2799<br>335.2956<br>343.1680<br>359.1629<br>471.2881
LPE(18:2)	477.2849	155.0104<br>182.0577<br>198.0526<br>221.2264<br>235.2420<br>245.2264<br>263.2369
LPE(20:0)	509.3481	155.0104<br>182.0577<br>198.0526<br>253.2890<br>267.3046<br>277.2890<br>293.2839
LPE(20:1)	507.3317	152.9958<br>182.0224<br>196.0380<br>214.0486<br>265.2901<br>291.2693<br>307.2643
Netilmicin	475.1306	160.0974<br>173.1290<br>175.1447<br>189.1239<br>191.1396<br>282.1818<br>299.2083
Polidocanol	582.3144	175.0970<br>185.1905<br>193.1076<br>211.2062<br>219.1232<br>229.2168
2-Methyl-5-(8,11,14-pentadecatrienyl)-1,3-benzenediol	328.0778	135.1174<br>137.0603<br>149.1330<br>151.0759<br>163.1487<br>165.0916<br>177.1643
Ethyl abietic acid	330.0264	83.0861<br>97.1017<br>119.0861

		133.1017<br>135.1174<br>149.1330<br>147.1174
2-hydroxyphytanic acid	328.2904	72.9931<br>253.2901<br>265.2901<br>281.2850<br>283.3006<br>309.2799<br>327.2905
Saussurea lactone	234.1616	125.0603<br>147.1174<br>149.1330<br>159.1174<br>161.1330<br>163.1487<br>173.1330
Menthone 8-thioacetate	228.1102	115.0218<br>117.0374<br>143.0531<br>149.0966<br>151.1123<br>155.0531<br>157.0323
Auxin b	310.1114	87.0446<br>101.0239<br>131.0344<br>179.1800<br>189.1643<br>205.1592<br>205.1956
Tocotrienol	424.3378	261.2224<br>275.2380<br>277.2537<br>289.2537<br>381.2799<br>383.2956<br>393.2799
Cloversaponin I	632.3911	145.0137<br>147.0293<br>149.0450<br>175.0243<br>193.0348<br>407.3314<br>409.3470
Astaxanthin	596.3856	231.1385<br>261.1643<br>269.1542<br>271.1698<br>281.1542<br>285.1855<br>295.1698
Inosine	268.0809	98.9847<br>108.0198<br>110.0354<br>122.0354<br>133.0150<br>135.0307<br>137.0463

Cer(d18:0/18:1)	565.5401	141.1638<br>155.1794<br>169.1951<br>183.2107<br>195.2107<br>211.2420<br>197.2264
3,7-Dihydroxy-5-cholan-24-Oic acid	392.2753	59.0139<br>329.2850<br>347.2956<br>355.2643<br>373.2748<br>391.2854
Glycocholic acid	465.3081	363.2905<br>389.2697<br>391.2854<br>402.3014<br>404.2806<br>418.2963<br>420.3119
Taurohyocholic acid	515.2915	106.9803<br>119.9755<br>121.9912<br>126.0225<br>124.0068<br>149.9861<br>152.0018
Tauroursodeoxycholic acid	499.2951	166.0174<br>329.2844<br>347.2950<br>357.2794<br>372.2903<br>373.2743<br>374.3059
Lithocholic acid taurine conjugate	483.2999	100.0035<br>102.0191<br>114.0191<br>116.0348<br>299.2739<br>313.2895<br>317.2844
Cholesteryl docosahexaenoic acid	696.5532	285.2588<br>309.2224<br>327.2330<br>367.3370<br>369.3163<br>369.3527<br>383.3319

Table S9. Pathway analysis details of altered metabolites in OS: Spoonbilss from Odiel Marshes, OG: Black-headed gulls from Odiel Marshes, TYG: yellow-legged gulls and TS: storks from La Tapa Saltworks. Match Status: the number of altered metabolites of the total metabolites involved in the route, with the p-value calculated from the enrichment analysis; Impact: pathway impact value calculated from pathway topology analysis.

Pathway Name (TYG vs TS)	Match Status	p	Impact
Taurine and hypotaurine metabolism	1/8	0.026898	0.0
Glycerolipid metabolism	1/16	0.053215	0.01246
Ether lipid metabolism	1/17	0.056464	0.08856
Biosynthesis of unsaturated fatty acids	1/36	0.11652	0.0
Glycerophospholipid metabolism	1/36	0.11652	0.12221
Fatty acid elongation	1/39	0.12572	0.0
Fatty acid degradation	1/39	0.12572	0.0
Tyrosine metabolism	1/42	0.13484	0.01174
Primary bile acid biosynthesis	1/46	0.14688	0.03799
Fatty acid biosynthesis	1/47	0.14987	0.01473
**Pathway Name (OS vs OG)**	**Match Status**	**p**	**Impact**
Primary bile acid biosynthesis	2/46	0.0089882	0.07598
Taurine and hypotaurine metabolism	1/8	0.026898	0.0
Sphingolipid metabolism	1/32	0.10414	0.21576
Steroid biosynthesis	1/41	0.13181	0.0
Purine metabolism	1/67	0.20791	0.00267

Table S10. Fecal altered metabolites ordered by subclass from comparing TYG and CLG groups. RT: retention time, FC: Fold change, IM: Ionization mode, TYG: yellow-legged gulls from La Tapa Saltworks and CLG: lesser black-backed from Cetina Saltsworks. Lysophosphatidylethanolamine (LPE); *p*-value obtained from one-way ANOVA followed by Tuckey Test and corrected by Benjamini-Hochberg multiple post-correction

Experimental mass (Da)	Theoretical mass (Da)	RT (min)	Compounds	[TYG] vs [CLG]		IM	Adduct	Subclass	Score (DB)
				FC	p-value				
269.2733	269.2718	13.31	Capsi-amide	0.13	0.0001	ESI+	$[M+H]^+$	Carboxylic acid derivatives	95.62
294.2193	294.2194	10.62	3,4-Dimethyl-5-pentyl-2-furanheptanoic acid	4.46	0.0244	ESI-	$[M-H]^-$	Fatty acids and conjugates	95.96
256.2410	256.2402	13.54	Palmitic Acid	0.80	0.0066	ESI-	$[M-H]^-$	Fatty acids and conjugates	97.6
308.2716	308.2715	14.41	11,14-Eicosadienoic Acid	0.37	0.0004	ESI+	[M+H]+	Fatty acids and conjugates	99.52
294.2197	294.2194	11.20	Colneleic acid	4.75	0.0005	ESI+	$[M+H]^+$	Fatty acids and conjugates	98.38
370.3088	370.3083	15.85	Docosanedioic acid	0.29	0.0001	ESI+	$[M+H]^+$	Fatty acids and conjugates	95.12
335.3205	335.3188	11.58	Allyl cyclohexylacetate	2.91	0.0346	ESI+	$[M+H]^+$	Fatty amides	98.87
453.286	453.2855	9.88	LPE(16:0)	0.46	0.0014	ESI-	$[M-H]^-$	Glycerophosphoethanolamines	95.96
578.3809	578.3818	11.40	5-spirostan-3-yl-D-glucoside	0.72	0.0149	ESI-	$[M-H]^-$	Steroidal glycosides	99.43
429.3244	429.3242	13.59	Imperialine	0.20	0.0459	ESI-	$[M-H]^-$	Steroidal alkaloids	95.58
515.2905	515.2916	8.35	Taurohyocholic acid	0.32	0.0074	ESI-	$[M-H]^-$	Bile acids, alcohols and derivatives	95.49

Table S11. List of fragments of the MS/MS experiment for the annotation of altered metabolites in the comparison of TYG and CLG groups. TYG: yellow-legged gulls from La Tapa Saltworks and (CLG) lesser black-backed from Cetina Saltsworks. Lysophosphatidylethanolamine (LPE).

Compounds	Exp. Mass (m/z)	Fragmentos (m/z)
Capsi-amide	269.2733	99.1168<br>111.1168<br>113.1325<br>125.1325<br>127.1481<br>139.1481<br>141.1638
3,4-Dimethyl-5-pentyl-2-furanheptanoic acid	294.2193	121.0653<br>123.0810<br>129.0916<br>163.1123<br>165.1279<br>175.1123<br>177.1279
Palmitic Acid	256.2410	57.0704<br>59.0133<br>71.0861<br>85.1017<br>99.1174<br>113.1330<br>127.1487
Colneleic acid	294.2197	67.0548<br>69.0704<br>71.0861<br>81.0704<br>83.0861<br>93.0704<br>95.0861
Docosanedioic acid	370.3088	199.1698<br>213.1855<br>227.2011<br>241.2168<br>251.2739<br>255.2324<br>263.2739
Allyl cyclohexylacetate	335.4405	67.0548<br>69.0704<br>71.0861<br>79.0548<br>81.0704<br>83.0497<br>83.0861
LPE(16:0)	453.286	122.9853<br>137.0009<br>140.0118<br>152.9958<br>182.0224<br>196.0380<br>211.2431
(25)-5-spirostan-3-yl-D-glucoside	578.3809	161.0450<br>179.0556

		285.2582<br>301.2531<br>303.2688<br>329.2481<br>341.2481
Taurohyocholic acid	515.2905	126.0225<br>149.9861<br>152.0018<br>166.0174<br>363.2899<br>345.2794<br>371.2586

Table S12. Pathway analysis details of altered metabolites in TYG: yellow-legged gulls from La Tapa Saltworks and (CLG) lesser black-backed from Cetina Saltsworks. Match Status: the number of altered metabolites of the total metabolites involved in the route, with the p-value calculated from the enrichment analysis; Impact: pathway impact value calculated from pathway topology analysis.

**Pathway Name (TYG vs CLG)**	**Match Status**	**p**	**Impact**
Ether lipid metabolism	1/17	0.034248	0.0
Biosynthesis of unsaturated fatty acids	1/36	0.07159	0.0
Fatty acid elongation	1/39	0.077396	0.0
Fatty acid degradation	1/39	0.077396	0.0
Tyrosine metabolism	1/42	0.083179	0.01174
Fatty acid biosynthesis	1/47	0.092762	0.01473

Table S13. Summary of the main altered metabolic pathways for each comparison.

	**Pathway analysis**
**OC vs CS**	➢ Sphingolipid metabolism<br>➢ Primary bile acid biosynthesis<br>➢ Fatty acid biosynthesis
**OS vs OG**	➢ Primary bile acid biosynthesis<br>➢ Sphingolipid metabolism<br>➢ Purine metabolism
**TYG vs TS**	➢ Glycerolipid metabolism<br>➢ Ether lipid metabolism<br>➢ Glycerophospholipid metabolism<br>➢ Tyroside metabolism<br>➢ Primary bile acid biosynthesis<br>➢ Fatty acid biosynthesis
**TYG vs CLG**	➢ Primary bile acid biosynthesis<br>➢ Sphingolipid metabolism<br>➢ Purine metabolism

Table S14. Coefficient of variation (CV) of gut metabolites calculated in quality control (QC) samples. Lysophosphatidylcholine (LPC), lysophosphatidylethanolamine (LPE),

phosphatidylethanolamine (PE); phosphatidylglycerol (PG); phosphatidylserine (PS); monoglyceride (MG).

**Compound**	**% CV in QC**<br>**OS vs CS**
10-hydroxystearic acid	4.99
2-hydroxy myristic acid	8.5
16-Hydroxy hexadecanoic acid	6.91
2-Dodecylbenzenesulfonic acid	5.26
3,6,7-Trihydroxy-5-cholanoic acid	4.96
3,7-Dihydroxy-5-cholanoic acid	8.97
5-Cyprinolsulfate	4.09
5-pregnan-3,20-dione	3.8
Cer(d18:1/12:0)	4.54
Epothilone C	6.44
Lithocholic acid glycine conjugate	8.37
LPE(20:0)	9.73
N-Undecylbenzenesulfonic acid	4.13
Palmitic Acid	7.05
Taurohyocholic acid	3.46
Tauroursodeoxycholic acid	5.25
PC(22:2/14:1)	8.92
Cer(d18:1/14:0)	9.98
Phytal	6.96
3',5'-Cyclic Inosine monophosphate (cIMP)	9.14
5,8-Epoxy-5,8-dihydro-3-hydroxy-8'-apo-b,y-carotenal	4.03
**Compound**	**% CV in QC**<br>**OS vs OG and TYG vs TS**

2-hydroxyphytanic acid	6.62
10-hydroxystearic acid	7.15
Menthone 8-thioacetate	5.75
12-Hydroxy-8,10-octadecadienoic acid	2.15
16-Hydroxy hexadecanoic acid	5.86
22-Hydroxydocosanoic acid	8.84
22-Oxo-docosanoate	6.77
2-Dodecylbenzenesulfonic acid	8.11
3,4-Dimethyl-5-pentyl-2-furanheptanoic acid	7.83
3,7-Dihydroxy-5-cholan-24-Oic acid	7.47
9,10-Dihydroxystearic acid	4.93
Ethyl abietic acid	7.29
Auxin b	6.86
Cer(d18:0/18:1)	7.37
Cloversaponin I	7.45
Colneleic acid	3.51
DG(15:0/18:4)	8.56
Dihydroabietic acid	9.52
Epothilone C	5.92
Glycocholic acid	9.44
Hexyl 3-mercaptobutanoate	4.87
Inosine	6.52
Lithocholic acid taurine conjugate	7.69
LPE(20:1)	5.91
Mucronine B	5.41
N2,N5-Dibenzoyl-L-ornithine	6.28
N-Undecylbenzenesulfonic acid	10.63

Octadecyl fumarate	7.86
PA(16:0/16:0)	4.34
Palmitic Acid	5.66
Saussurea lactone	7.35
Stearolic acid	4.16
Sterculic acid	5.74
Taurohyocholic acid	3.49
Tauroursodeoxycholic acid	8.87
9-Hexadecenoate	8.06
Cholesteryl docosahexaenoic acid	4.51
2-Methyl-5-(8,11,14-pentadecatrienyl)-1,3-benzenediol	2.15
3',4'-Dihydrodiol	5.86
Astaxanthin	9.17
Capsi-amide	1.93
Docosanedioic acid	6.77
Edetate	10.48
Imperialine	7.57
LPC(18:0)	4.67
LPE(18:0)	3.88
Palmitic amide	3.23
PC(22:2/14:1)	6.62
Polidocanol	8.98
Zinnolide	8.61
**Compound**	**% CV in QC TYG vs CSG**
5-spirostan-3-yl-D-glucoside	5.55

3,4-Dimethyl-5-pentyl-2-furanheptanoic acid	7.1
Asparagoside B	4.52
Dihydroabietic acid	3.19
Imperialine	4.97
Isoleucyl-Phenylalanine	5.56
Leucyl-leucine	4.2
LPE(16:0)	1.74
Palmitic Acid	0.68
Taurohyocholic acid	7.6
11,14-Eicosadienoic Acid	6.09
Allyl cyclohexylacetate	10.85
Capsi-amide	9.93
Cinitapride	2.59
Colneleic acid	4.19
Docosanedioic acid	8.15
Palmitic amide	8.24

Figure S1. Location of the study zones.

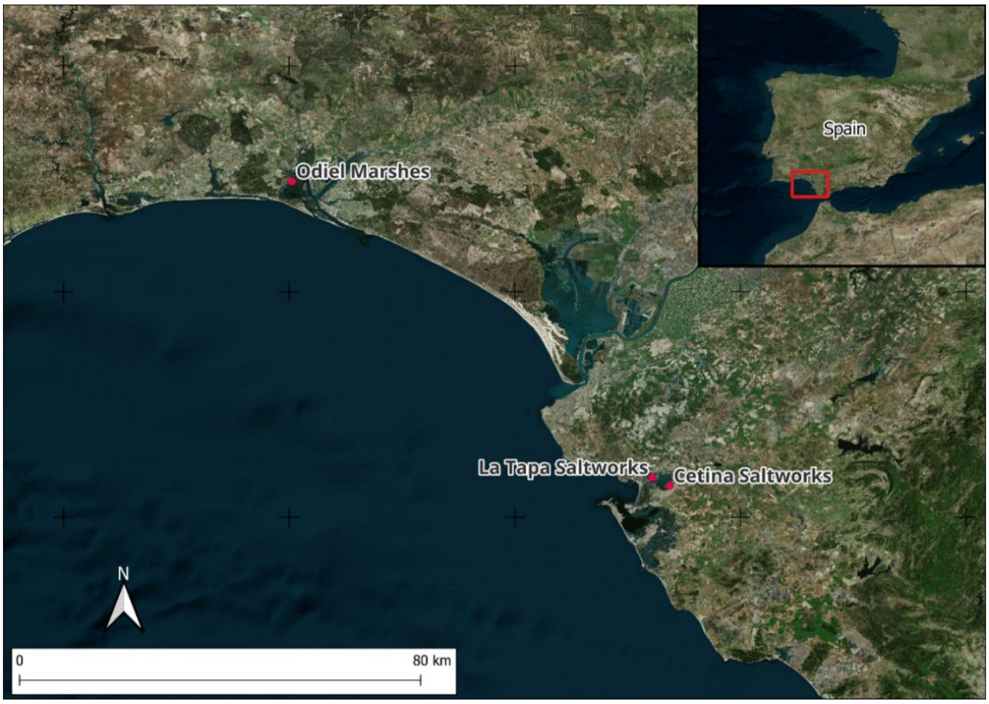

Figure S2. The figure highlights the main classes of metabolites that were altered between the groups of a) OS and OG groups and b) TS and TYG groups

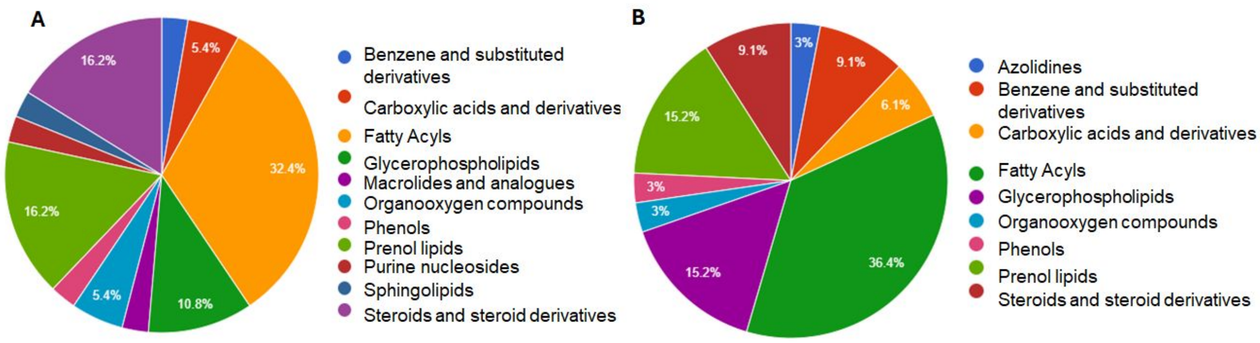

Figure S3. The figure illustrates the metabolic pathways affected by these alterations in a) OS and OG groups and b) TS and TYG groups

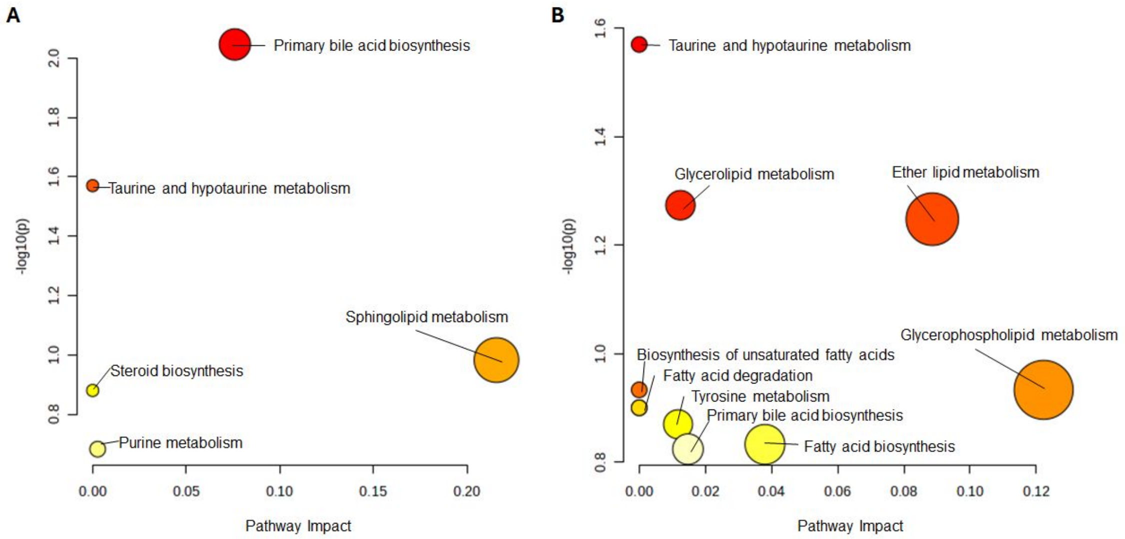

Figure S4. Typical fecal metabolome profiling of waterfowl using UHPLC-QTOF-MS analysis in a) positive and b) negative ionization modes.

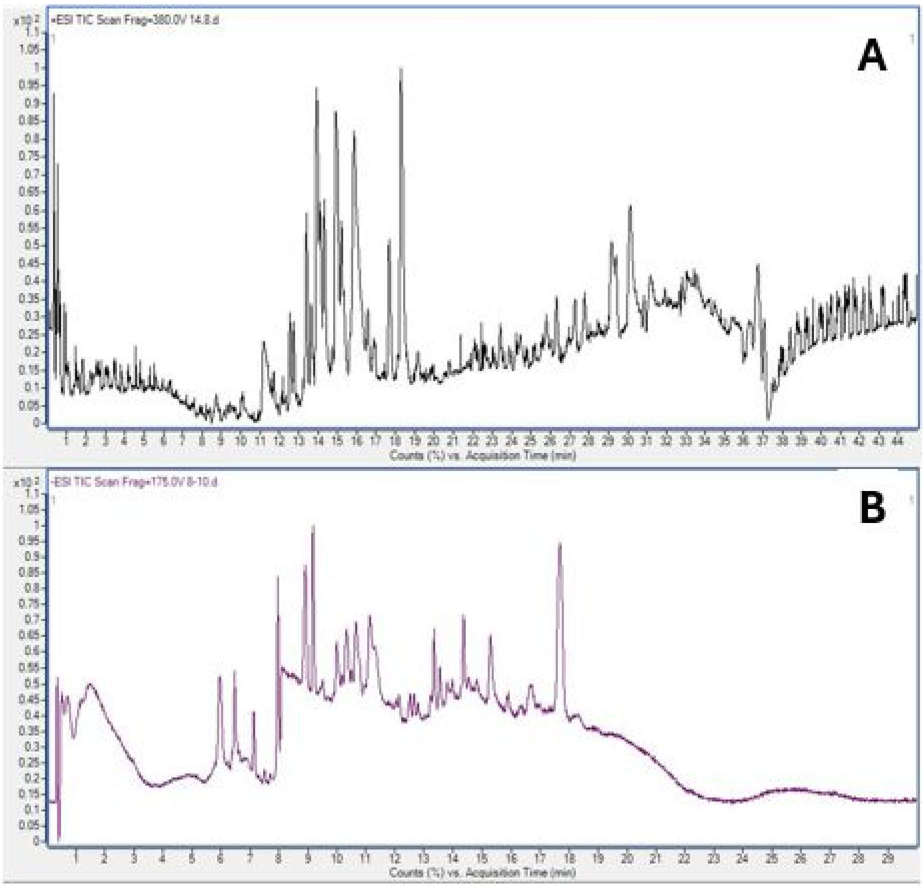

Figure S5. The PLS-DA plots demonstrated good separation between groups for OS and CS groups in A) positive mode and B) negative modes; OS vs OG groups in C) positive mode and D) negative modes; TYG and TS groups in E) positive mode and F) negative modes; and TYG and CLG groups in G) positive mode and H) negative modes.

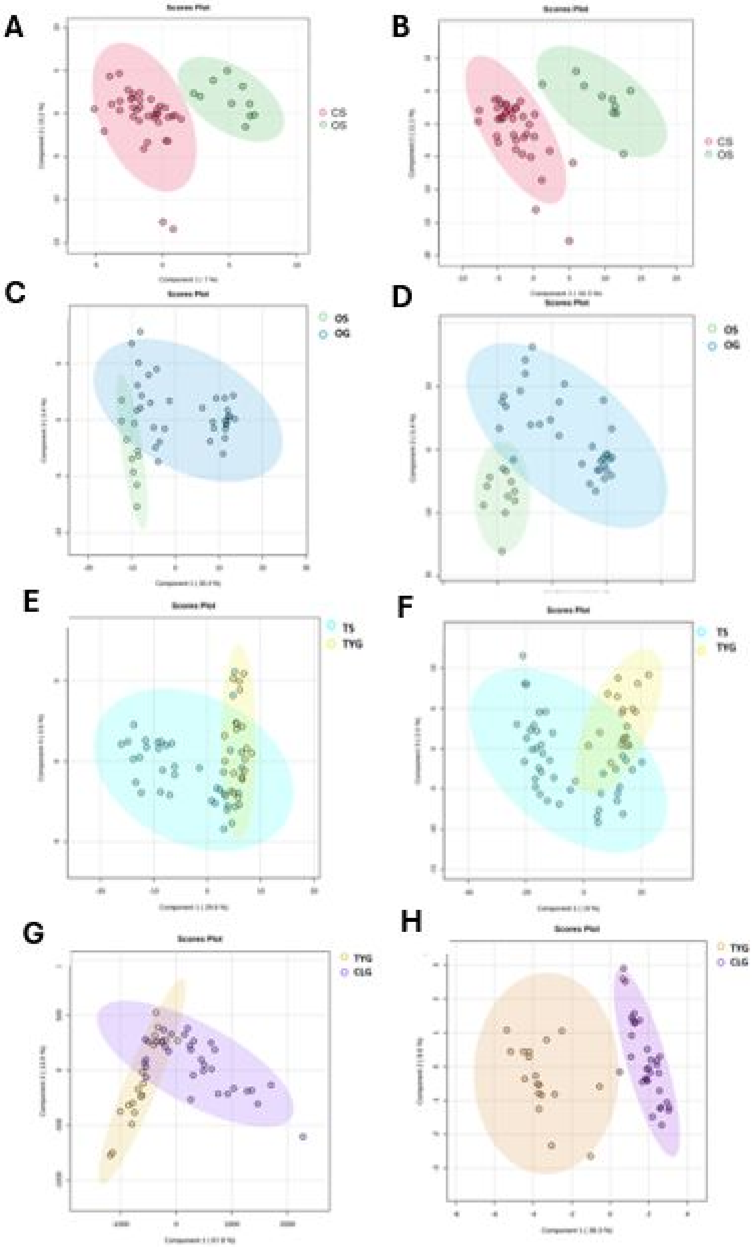

Figure S6. The PCA plots demonstrated good clustering of the quality control samples for OS and CS groups in A) positive mode and B) negative modes; OS vs OG groups in C) positive mode and D) negative modes; TYG and TS groups in E) positive mode and F) negative modes; and TYG and CLG groups in G) positive mode and H) negative modes.

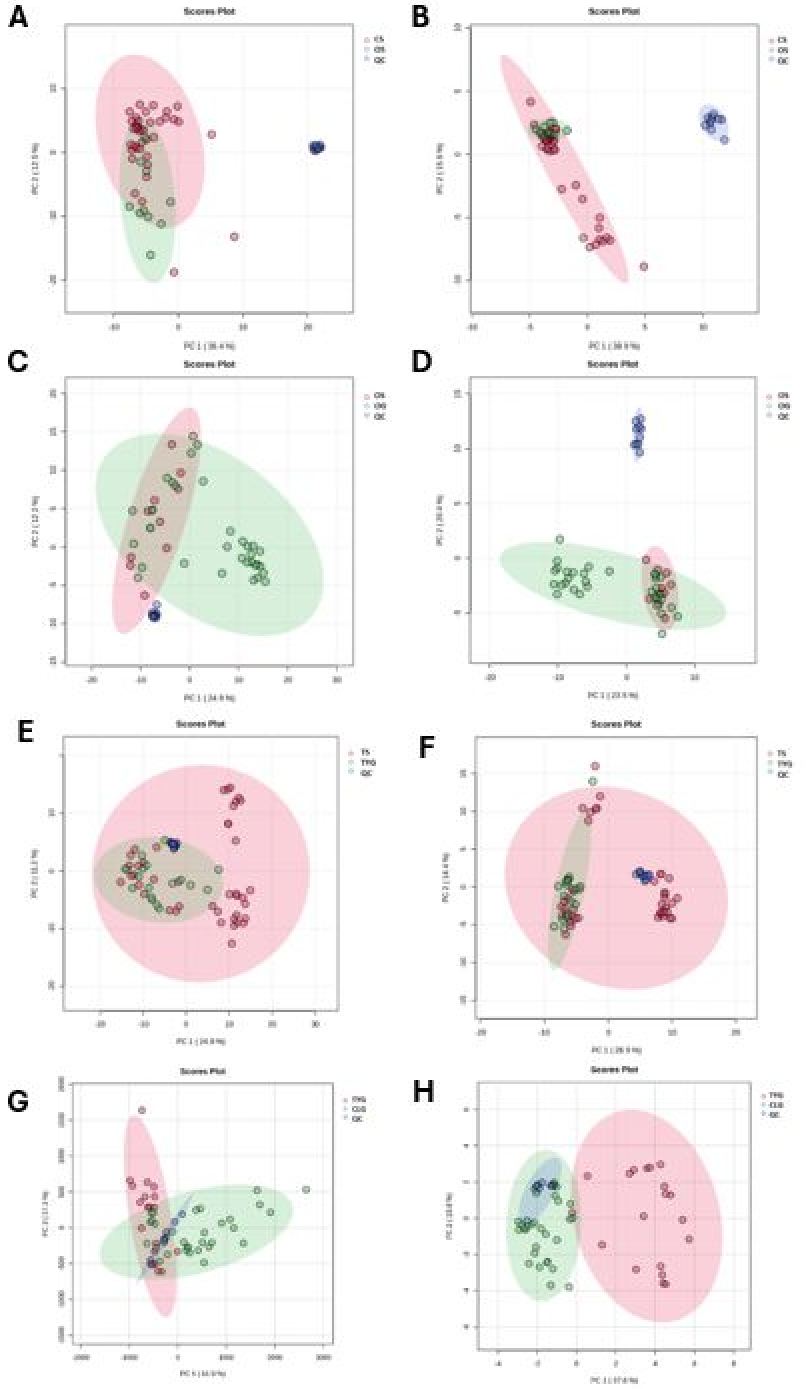

Figure S7. Blank samples from (a) UHLC-ESI(+)-QTOF-MS (b) UHPLC-ESI(-)-QTOF-MS.

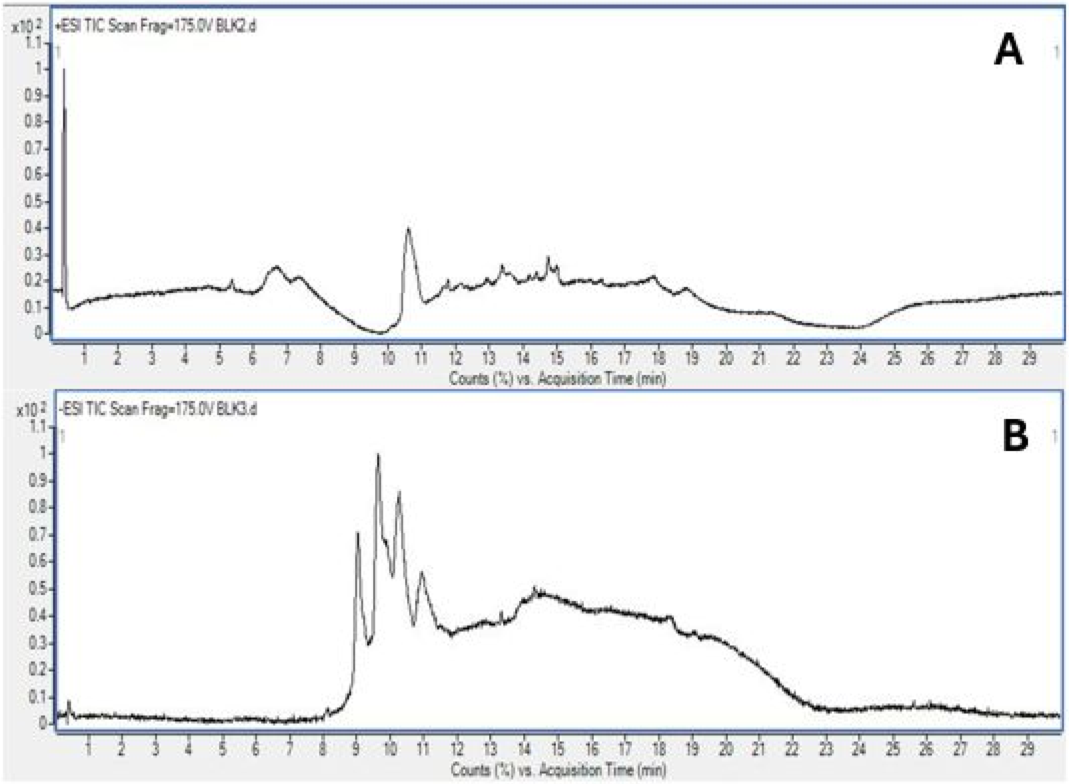

Supplement: Supplementary file 1 [file ew5c01312_si_001.pdf]
